# Quantitative Disease Resistance under Elevated Temperature: Genetic Basis of New Resistance Mechanisms to *Ralstonia solanacearum*

**DOI:** 10.3389/fpls.2017.01387

**Published:** 2017-08-22

**Authors:** Nathalie Aoun, Laetitia Tauleigne, Fabien Lonjon, Laurent Deslandes, Fabienne Vailleau, Fabrice Roux, Richard Berthomé

**Affiliations:** LIPM, Centre National de la Recherche Scientifique, Institut National de la Recherche Agronomique, INPT, Université de Toulouse Castanet-Tolosan, France

**Keywords:** climate warming, quantitative disease resistance, *Ralstonia solanacearum*, *Arabidopsis thaliana*, natural diversity, genome wide association mapping

## Abstract

In the context of climate warming, plants will be facing an increased risk of epidemics as well as the emergence of new highly aggressive pathogen species. Although a permanent increase of temperature strongly affects plant immunity, the underlying molecular mechanisms involved are still poorly characterized. In this study, we aimed to uncover the genetic bases of resistance mechanisms that are efficient at elevated temperature to the *Ralstonia solanacearum* species complex (RSSC), one of the most harmful phytobacteria causing bacterial wilt. To start the identification of quantitative trait loci (QTLs) associated with natural variation of response to *R. solanacearum*, we adopted a genome wide association (GWA) mapping approach using 176 worldwide natural accessions of *Arabidopsis thaliana* inoculated with the *R. solanacearum* GMI1000 strain. Following two different procedures of root-inoculation (root apparatus cut vs. uncut), plants were grown either at 27 or 30°C, with the latter temperature mimicking a permanent increase in temperature. At 27°C, the *RPS4/RRS1-R* locus was the main QTL of resistance detected regardless of the method of inoculation used. This highlights the power of GWA mapping to identify functionally important loci for resistance to the GMI1000 strain. At 30°C, although most of the accessions developed wilting symptoms, we identified several QTLs that were specific to the inoculation method used. We focused on a QTL region associated with response to the GMI1000 strain in the early stages of infection and, by adopting a reverse genetic approach, we functionally validated the involvement of a strictosidine synthase-like 4 (SSL4) protein that shares structural similarities with animal proteins known to play a role in animal immunity.

## Introduction

Global changes are predicted to increase the frequency of extreme climate events and to accentuate abiotic stresses such as temperature increase, drought, and water logging (Orlowsky and Seneviratne, 2012; IPCC, 2014), thereby impacting crop development (e.g., rate of photosynthetic carbon assimilation, rate of leaf initiation, leaf expansion, root architecture, or reproduction) and ultimately yield (Hatfield et al., 2011; Gray and Brady, 2016). Amongst climatic risks, global surface temperature is predicted to rise by the end of the century, from 1.0 up to 4.8°C, with higher frequencies and longer periods of heat waves (IPCC, 2014). While an increase of mean temperature constitutes one of the major abiotic stresses that plants have to cope with (Bita and Gerats, 2013; Suzuki et al., 2014), it is also expected to favor the emergence of new pathogens and to increase the occurrence of epidemics (Garrett et al., 2006; Evans et al., 2008; Bebber et al., 2013). For example, recent studies reported that climate change likely increased the emergence of highly aggressive and invasive strains of rust *Puccinia graminis, P. striiformis*, and of the oomycete *Phytophthora infestans* (Hovmoller et al., 2008; Singh et al., 2011; Cooke et al., 2012; Bebber et al., 2013). These future outbreaks are a major concern for the maintenance of global food security. Therefore, identifying and studying the genetic and molecular basis of defense mechanisms allowing plants to cope with epidemics under higher temperature conditions is critical.

In their natural habitats, plants have evolved complex defense responses to deal with the simultaneous and/or sequential attacks from various bio-aggressors (e.g., viruses, bacteria, fungi, oomycetes, and herbivores) (Roux and Bergelson, 2016). Defense responses include plant preformed physical or chemical barriers (e.g., rigid cell wall, presence of cuticles or trichomes, production of toxic or repellent compounds) (Osbourn, 1996; Nurnberger and Lipka, 2005) as well as immune signaling responses that are activated upon pathogen recognition. This latter type of resistance mechanism corresponds to a two-level defense system described as the zig-zag model (Jones and Dangl, 2006; Dodds and Rathjen, 2010). In the first level, microbial elicitors, called pathogen- or microbe-associated molecular patterns (PAMPs or MAMPs) are perceived by plant cell surface and transmembrane pattern recognition receptors (PRRs) to initiate a signaling cascade leading to the MAMP- or PAMP-triggered immunity (MTI/PTI), that is efficient against a broad spectrum of pathogens. To overcome PTI, pathogens produce virulence factors called effectors which can promote effector-triggered susceptibility (ETS) by interfering with host defense responses. In turn, effectors can be specifically recognized by plant intracellular resistance proteins, known as nucleotide binding site-leucine rich repeat containing proteins (NLRs), thereby activating a second level of plant defense, the effector-triggered immunity (ETI). ETI results in a strong defense response often associated with a hypersensitive response (HR), characterized by a rapid and local cell death. However, this type of qualitative response is generally specific to a single pathogen species, and even strain-specific, thereby leading to a strong selection of virulent strains that can bypass ETI (Roux et al., 2014). Another form of resistance called Quantitative Disease Resistance (QDR), is characterized by a reduction rather than an absence of disease (St Clair, 2010; Mundt, 2014; Roux et al., 2014; French et al., 2016) and is typically polygenic (Poland et al., 2009). Knowing that QDR provides a durable and a broad-spectrum resistance, this form of resistance appears to be unsurprisingly much more prevalent than ETI in crops and natural plant populations (Young, 1996). The characterization of the main molecular mechanisms underlying PTI and ETI have paved the way to decipher basal and specific immune responses, which is not currently the case of QDR's molecular mechanisms that remain largely unknown (Roux et al., 2014).

Plants are exposed to multiple abiotic and biotic stresses either in crop fields or in natural populations. However, studies deciphering the molecular mechanisms governing plant response to these combined stresses are scarce (Mittler, 2006; Suzuki et al., 2014). Yet, several transcriptome studies recently demonstrated that despite a certain overlap, each combination of stress involves a unique response that could not be easily predicted by the study of each stress individually, and leading to either positive or negative effects on host immunity (Atkinson and Urwin, 2012; Atkinson et al., 2013; Prasch and Sonnewald, 2013; Rasmussen et al., 2013; Suzuki et al., 2014; Onaga et al., 2017). Remarkably, an increase in temperature (3–7°C) was demonstrated (i) to inhibit several major defense mechanisms induced following a pathogen attack, regardless of the pathogen and the plant species considered, or (ii) to suppress ETI-HR related phenotypes. For example, in *Arabidopsis thaliana*, the expression of two regulators of plant immunity, *PAD4* and *EDS1*, is down-regulated by a rise in temperature from 22 to 28°C (Yang and Hua, 2004). RPS4- or RPM1-mediated resistance responses to *Pseudomonas syringae* pv. *tomato* (*Pst*) DC3000 strains are efficient at 22°C but inhibited at 28°C (Wang et al., 2009). In addition, the HR triggered either by *Pst* DC3000 strain containing the HopZ1A avirulent effector or by the *RPW8* gene conferring resistance against powdery mildew is suppressed by a temperature above 28 and 30°C, respectively (Xiao et al., 2003; Menna et al., 2015). However, for *Pst* DC3000 strains carrying the *AvrRpt2* effector gene, contradictory results were observed. Indeed, elevated temperatures led to either a resistance inhibition (Wang et al., 2009) or a HR suppression without ETI-mediated virulence suppression (Menna et al., 2015). Similar examples of inactivation by an elevated temperature were also reported in other plant species. Resistance conferred by the *Mi-1* gene to root-knot nematodes, by the *N* gene to tobacco mosaic virus (TMV) and by the *Cf4* and *Cf9* genes to the fungal pathogen *Cladosporium fulvum* are inactivated in tomato at temperatures above 28, 30, and 33°C, respectively (Whitham et al., 1996; Hwang et al., 2000; de Jong et al., 2002). An elevated temperature also negatively modulated plant defense response linked to *Rp1-D21* in maize (Negeri et al., 2013). By contrast, partial or more efficient resistances related to *Yr36, Xa7*, and *Pi54* genes were also described against *P*. *graminis* in wheat (Fu et al., 2009), *Xanthomonas oryzae* pv. *oryzae* and *Magnaporthe oryzae* in rice, respectively (Webb et al., 2010; Onaga et al., 2017). Although an increase of temperature can differentially balance host immunity, the molecular mechanisms involved remain elusive.

The *Ralstonia solanacearum* species complex (RSSC) is responsible for the bacterial wilt, one of the most harmful disease causing tremendous yield losses in more than 200 plant species in tropical, subtropical, and warm temperate areas worldwide (Elphinstone, 2005). The strains composing the RSSC are phylogenetically represented by four phylotypes according to their geographical origin (I: Asia, II: America, III: Africa, IV: Australia-Indonesia-Japan) (Ailloud et al., 2015). The ability of RSSC to quickly adapt to new host plants or to the universal resistant reference Hawaii7996 tomato cultivar (Wicker et al., 2007) is thought to be promoted by (i) many molecular determinants involved in pathogenicity and host-range specificity, (ii) large and variable repertoires of type III effectors (T3Es), and (iii) a high evolutionary ability (Coupat et al., 2008; Remenant et al., 2010; Genin and Denny, 2012). In addition, climate warming has been recently proposed to be involved in the expansion of the potato brown rot disease caused by some RSSC strains in Bolivia (Castillo and Plata, 2016). Host resistance to RSSC, which remains the most efficient strategy of disease control, is generally controlled by a polygenic architecture (Mangin et al., 1999; Wang et al., 2000; Carmeille et al., 2006). In the model plant *A. thaliana*, the two molecular resistance mechanisms identified so far involve the LRR receptor-like kinase ERECTA (Godiard et al., 2003) and the RPS4/RRS1-R pair of immune receptors (Deslandes et al., 2002; Le Roux et al., 2015), respectively. Recently, Le Roux et al. (2015) demonstrated that RPS4 with RRS1-R represent a DNA-bound immune receptor complex with an integrated effector decoy that directly converts the virulence activity of the PopP2 effector into activation of immunity (Le Roux et al., 2015). Interestingly, this immune-receptor pair also confers resistance to various pathogens including the fungus *Colletotrichum higginsianum, Pst* DC3000 delivering the AvrRps4 effector, and the strain *CFBP6943* of *Xanthomonas campestris* pv. *campestris* (*Xcc*) (Narusaka et al., 2009; Debieu et al., 2016). In addition, in contrast to the well-characterized resistance against *R. solanacearum* GMI1000 strain identified in the *A. thaliana* accession Nd-1 (Deslandes et al., 1998, 2002), a tolerant phenotype to *R. solanacearum* BCCF402 strain in the *A. thaliana* Kil-0 accession, resulting in the absence of symptom despite a high bacterial multiplication *in planta*, was demonstrated to be also dependent on the PopP2 effector but with a specific allele of *RRS1-R* (Van der Linden et al., 2013). Thus, these results suggest that RRS1-R may also be involved in a QDR mechanism in the *A. thaliana* Kil-0 accession.

Despite the fact that a temperature increase is known to affect host resistance to *R. solanacearum* in several plant species (Hayward, 1991; Prior et al., 1996), no mechanism of resistance that is efficient under a permanent increase in temperature has been identified yet. Moreover, studies reporting the genetic architecture and the molecular mechanisms underlying the genetic diversity of plant responses to RSSC are still lacking in the context of climate change. Therefore, exploring natural genetic variation can help to identify uncovered sources of resistance to RSSC that are efficient under elevated temperature conditions. Thanks to the development of Next-Generation Sequencing (NGS) technologies and appropriate statistical methods (Bergelson and Roux, 2010), the method of genome-wide association (GWA) mapping recently emerged in plants and has been used so far in 11 species to fine map genomic regions associated with natural variation of response to a range of microbial enemies, including bacteria, fungi, and oomycetes (Bartoli and Roux, 2017). Notably, five quantitative trait loci (QTLs) identified by GWA mapping have been subsequently functionally validated in *A. thaliana*, demonstrating the power of this method to precisely dissect the intraspecific genetic variation underlying pathogen resistance (Bartoli and Roux, 2017). Therefore, in this study, we adopted a GWA mapping approach to fine map QTLs associated with natural variations of response to the *R. solanacearum* GMI1000 strain among 176 worldwide natural accessions of *A. thaliana*. Two inoculation methods were used and root-inoculated plants were incubated at two different temperatures to mimic a permanent increase of temperature. Whatever was the method of inoculation used, GWA mapping confirmed the *RPS4/RRS1-R* locus as the main QTL of resistance detected at 27°C, demonstrating the power of this approach to functionally fine map important loci for resistance to *R. solanacearum*. At 30°C, most accessions developed wilting symptoms, confirming the drastic effect of the temperature increase (+3°C) on the mechanisms of resistance triggered by plants at 27°C. Interestingly, we detected several QTLs, underlying natural variation for early plant defense response to *R. solanacearum* at 30°C. A reverse genetic approach revealed that one of these specific QTLs at 30°C involves a strictosidine synthase-like 4 (SSL4) protein.

## Materials and methods

### Bacterial strain, plant material, and growth conditions

The wild type *R. solanacearum* GMI1000 strain used in all inoculation experiments was grown in complete BG medium as described by Plener et al. (2010). A collection of 176 *A. thaliana* worldwide accessions was used in this study (Table S1). Five to 10 seeds of each accession were directly sown on Jiffy pots (Jiffy Products International AS, Norway) and stratified for 48 h at 4°C in order to release seed dormancy. Accessions were then grown in a growth chamber under controlled conditions for 4 weeks [22°C, 70% relative humidity (RH), 9 h of light] prior to phenotyping experiments. The 38 T-DNA insertion mutants (Ws-0 and Col-0 background) corresponding to 21 genes included in a 80 kb genomic region underlying a QTL of early plant defense response to *R. solanacearum* were identified using the online Arabidopsis gene mapping tool T-DNA express (http://signal.salk.edu/cgi-bin/tdnaexpress) (Table S2) and ordered from the Nottingham Arabidopsis Stock center (http://arabidopsis.info/). Corresponding seeds were sown and stratified as described above and grown in greenhouse conditions (26.5 ± 1.5°C, 16 h light). Progenies of genotyped homozygous of each mutant were harvested and grown for 4 weeks as described above before inoculation. The Col-0 accession (N60000), susceptible to the *R. solanacearum* GMI1000 strain, was used as a control in all experiments.

### Plant inoculation and phenotyping

Four-week-old plants were used in all experiments. Plant response to *R. solanacearum* GMI1000 strain was assessed at 27 and 30°C using two inoculation conditions: (i) the UNCUT condition previously described (Lohou et al., 2014), where the roots were not wounded thereby mimicking natural infection, and (ii) the CUT condition (Deslandes et al., 1998) where the roots were sectioned with scissors, ~1 cm from the bottom of the Jiffy pot, giving the bacteria a direct access to the xylem vessels. Plants were soaked for 15 min in 2 L per tray of a bacterial suspension at 1.10^7^ bacteria/mL and 1.10^8^ bacteria/mL, for the CUT and the UNCUT conditions, respectively. Inoculated plants were then transferred in growth chambers with controlled conditions at 27 or at 30°C (75% HR, 12 h light, 100 μmol m^−2^ s^−1^). The wilting symptoms were scored on an established 0 to 4 disease index scale (Deslandes et al., 1998) with the score 0 and 4 corresponding to healthy and dead plants, respectively. Symptoms were monitored from 3 to 13 days after inoculation (dai), and from 3 to 10 dai for plants incubated at 27 and 30°C, respectively.

### Natural variation of QDR

#### Experimental design

For each “CUT condition x temperature treatment” combination, an experiment of 1,152 plants was set up using a randomized complete block design (RCBD) of two experimental blocks. Each block was represented by six trays of 96 positions. Each block corresponded to 576 plants with three replicates per accession (*n* = 528 = 176 accessions ^*^ three replicates) and the control accession Col-0 placed in the same five positions within each tray (*n* = 30 = 6 trays ^*^ five replicates). In each block, the remaining 18 positions in the trays were left empty.

For each “UNCUT condition × temperature treatment” combination, an experiment of 570 plants was set up using a RCBD of three experimental blocks. Each block was represented by three trays of 64 positions. Each block corresponded to 192 plants with one replicate per accession (*n* = 176) and the control accession Col-0 placed in 14 positions across the three trays (*n* = 14). In each block, the remaining two positions in the trays were left empty.

#### Statistical analyses

For each “CUT/UNCUT condition × temperature treatment” combination, we used the following mixed model (MIXED procedure in SAS9.3; SAS Institute Inc., Cary, NC, USA) to explore the natural genetic variation of the disease index at each time point of phenotyping:

(1) $$\begin{array}{l} \text{disease }{\text{index}}_{ijc}=\mu +{\text{block}}_{i}+{\text{accession}}_{j}+{\text{block}}_{i}{\text{xaccession}}_{j}\\ \;+{\text{covCol}}_{c}+\varepsilon_{ijc} \end{array}$$

where μ is the overall mean of the phenotypic data, “block” accounts for differences in micro-environmental conditions between the two or three experimental blocks; “accession” corresponds to the genetic differences among the natural accessions; “block × accession” accounts for genetic differences among the natural accessions depending on the block; covCol is a covariate accounting for tray effects within blocks (phenotypic mean of the four or five Col-0 replicates per tray was used as a covariate); and “ε” is the residual term. Normality of the residuals was not improved by transformation of the data. The factor “block” was treated as a random factor, whereas the factor “accession” was treated as a fixed factor. Significance of the random effect was tested with likelihood ratio tests of models with and without this effect. Least-square means (LSmeans) were obtained for each natural accession and were subsequently used for GWA mapping analyses. Broad-sense heritabilities (*H*^2^) at each time point of phenotyping were estimated from the mean square (MS) of equation (1) using a formula adapted from Gallais (1990). Due to the absence of Col-0 control plants in some trays of the “UNCUT condition × 27°C” combination, the term “covCol_*c*_” was not modeled in Equation (1).

#### GWA mapping

The 176 natural accessions used in this study have been genotyped for 214,051 SNPs evenly spaced across the genome (Horton et al., 2012). In order to fine map the genomic regions associated with natural disease index variation at each time of phenotyping for each “CUT/UNCUT condition × temperature treatment” combination, we ran a mixed model implemented in the software EMMAX (Efficient Mixed-Model Association eXpedited; Kang et al., 2010). This mixed model includes a genetic kinship matrix *K* based on the 214,051 SNPs as a covariate to control for population structure in the mapping panel. Because of bias due to rare alleles, we only considered SNPs with minor allele relative frequency (MARF) > 10% (Brachi et al., 2010; Kang et al., 2010). Manhattan plots illustrating the results of phenotype-genotype associations at all stages of infection at 27 and 30°C, in the CUT and UNCUT conditions of inoculation are presented in Figures S1–S4, respectively. Corresponding Q-Q plots and lists of the most significantly associated SNPs (i.e., top SNPs with a –log_10_ *p*-value > 4) are depicted in Figures S5, S6 and Table S3.

#### Gene ontology and biological pathways enrichment tests

To determine the biological processes involved in response to *R. solanacearum* GM1000 strain at 30°C and perform comparisons between the two inoculation methods used, we first tested for each “CUT/UNCUT condition × time point of phenotyping” whether SNPs in the 0.1% upper tail of the –log_10_ *p*-value distribution were over-represented in each of 736 Gene Ontology Biological Processes from the GOslim set (Consortium, 2008). A total of 10,000 permutations were run to assess significance using the same methodology as described in Hancock et al. (2011) (Table S4). For each significant enriched biological process at a *P* < 0.05, we then retrieved the identity of all the genes containing SNPs in the 0.1% upper tail of the –log_10_ *p*-value distribution (Table S5). Finally, each list of genes, corresponding to each phenotyping time point, was used after removal of duplicates, to identify biological pathways significantly over-represented (*P* < 0.01) with the classification superviewer tool on the university of Toronto website (http://bar.utoronto.ca/ntools/cgi-bin/ntools_classification_superviewer.cgi) using the MAPMAN classification (Provart and Zhu, 2003; Tables S6, S7).

### T-DNA insertion mutants' validation, plant assays, and statistical analyses

For each of the 38 T-DNA insertion mutants located in a QTL region of early *A. thaliana* defense responses to *R. solanacearum* (see *Results* section), 12 seedlings were genotyped to check the presence of the T-DNA insertion and to identify homozygous plants. For each seedling, one leaf was collected and used for genomic DNA extraction was adapted from QIAGEN DNeasy kit®as described in Mayjonade et al. (2016). For genotyping, primer pairs were designed using the T-DNA primer design online tool (http://signal.salk.edu/tdnaprimers.2.html). All the primer sequences and corresponding PCR fragment sizes are listed in Table S2. For one PCR reaction, 2 μL of genomic DNA (10 ng/μL) were added to a PCR master mix composed of: 1 μL (10 pM) of each primer composing the LP+RP or RP+BP primer pairs (see Table S2), 0.5 μL (10 mM) dNTPs, 0.2 μL (10 μ/μL) of GoTaq® DNA polymerase (Promega, Madison, WI, USA), 5 μL of 5X GoTaq buffer and 16.2 μL of sterilized water. The PCR cycling conditions were as follow: 95°C for 2 min; 10 cycles at 95°C for 30 s, 62 to 52°C for 30 s (touch-down, 1°C decrease at each cycle) and 72°C for 1 min; 30 cycles at 95°C for 30 s, 52°C for 30 s, and 72°C for 1 min; 72°C for 2 min.

T-DNA insertion mutants were inoculated using the CUT inoculation method with a bacterial suspension of 1.10^7^ bacteria/mL and transferred at 30°C. Three to six independent experiments were made for each T-DNA insertion mutant. In all experiments, plants were organized according to a RCBD. To test whether the disease index was statistically different between the wild type Col-0 and each T-DNA mutant, we used a Kruskal-Wallis analysis under the *R* environment version 3.3.2 (R_Development_Core_Team, 2013). The dynamics of T-DNA mutant lines response to the *R. solanacearum* GMI1000 strain was drawn using ggplot2 package (http://ggplot2.org/) showing the confidence interval.

### RNA extractions and RT-qPCR

RNA extractions and RT-qPCR analyses were performed as previously described (Le Roux et al., 2015) using two leaves from healthy plants. Primer pairs used are listed in Table S8.

## Results

### Extensive genetic variation among worldwide accessions of *A. thaliana* for the response to *R. solanacearum*: effects of temperature and inoculation methods

**Figure 1:**
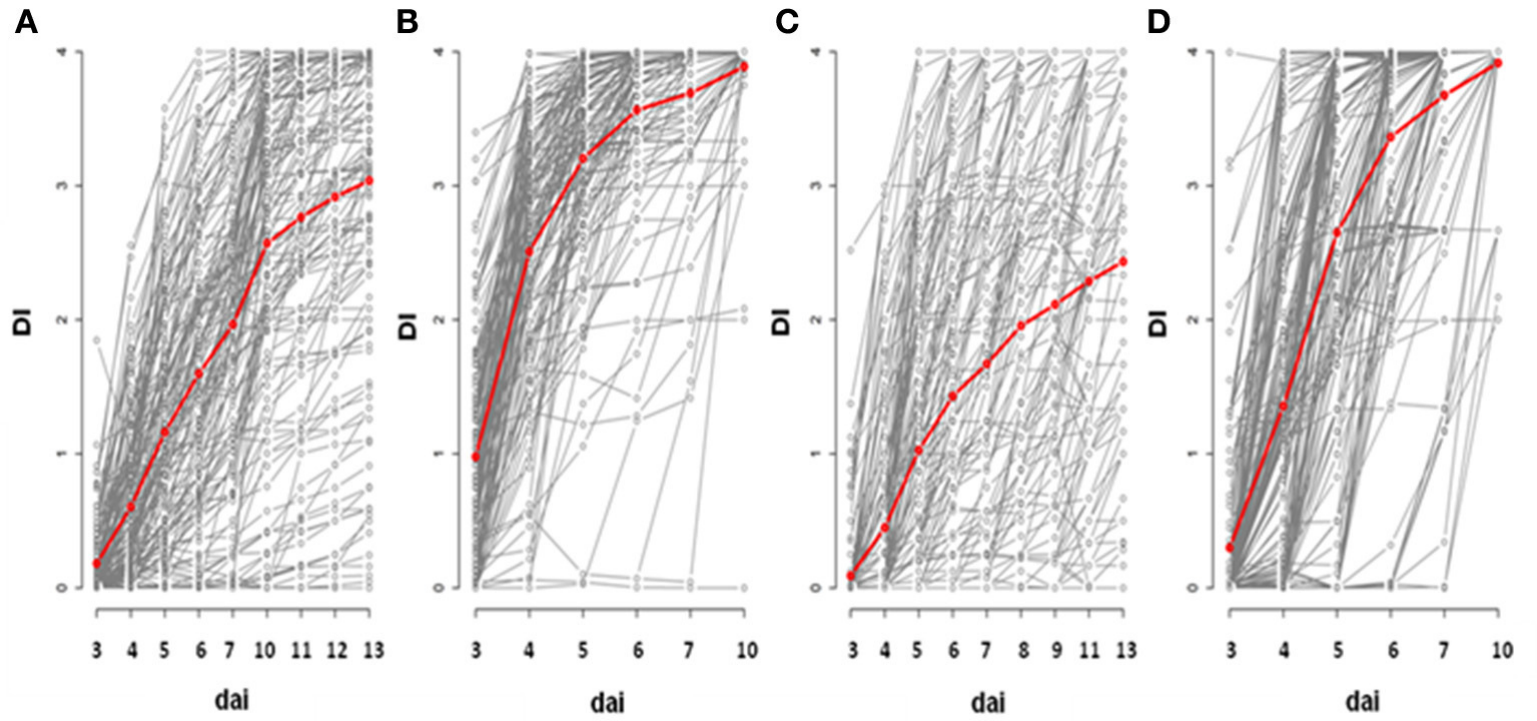
Genetic diversity of plant response to *R. solanacearum* GMI1000 strain in the CUT and UNCUT conditions of inoculation at 27 and 30°C. The red line represents the mean of disease index over all the accessions. Inoculation was performed at 27°C **(A,C)** and 30°C **(B,D)** on 4-week-old plants for which roots have been cut **(A,B)** or left uncut **(C,D)**. dai, days after inoculation; DI, disease index.

Based on the phenotyping of 176 worldwide accessions of *A. thaliana*, we detected substantial genetic variation in the response to the GM1000 strain for each “temperature treatment × inoculation method” (Figure 1; Tables 1, 2), with the presence of resistant accessions to *R. solanacearum* at 30°C (Figures 1B,D). The high broad-sense heritability estimates observed at all stages of infection suggests that plant response to *R. solanacearum* is genetically controlled even at early stages of infection (Figure 1; Tables 1, 2). In both inoculation methods, accessions were on average more susceptible at 30°C compared to 27°C (CUT – 10 dai, *F* = 136.9, *P* < 0.0001; UNCUT – 7 dai, *F* = 227.1, *P* < 0.0001) (Figure 1). At 27°C, accessions were on average more susceptible in the CUT condition than in the UNCUT condition (13 dai, *F* = 17.0, *P* < 0.0001) (Figure 1). At 30°C, although the accessions had on average a similar disease index at the late stages of infection (6 dai: *F* = 3.85, *P* = 0.0509; 7 dai: *F* = 0.02, *P* = 0.8889; 10 dai: *F* = 0.89, *P* = 0.3472), the dynamics of disease induction was faster in the CUT condition compared to the UNCUT condition at the early stages of infection (3 dai: *F* = 58.7, *P* < 0.0001; 4 dai: *F* = 88.7, *P* < 0.0001; 5 dai: *F* = 17.9, *P* < 0.0001) (Figure 1).

**Table 1:** Natural variation among natural accessions for disease index in the CUT condition.

**A. 27°C—CUT**																		
**Traits**	**Symptoms D3**		**Symptoms D4**		**Symptoms D5**		**Symptoms D6**		**Symptoms D7**		**Symptoms D10**		**Symptoms D11**		**Symptoms D12**		**Symptoms D13**	
**Model terms**	***F*** **or LRT**	***P***	***F*** **or LRT**	***P***	***F*** **or LRT**	***P***	***F*** **or LRT**	***P***	***F*** **or LRT**	***P***	***F*** **or LRT**	***P***	***F*** **or LRT**	***P***	***F*** **or LRT**	***P***	***F*** **or LRT**	***P***
*Block*	1.70	0.1929	0.00	1.0000	1.30	0.2542	23.90	<0.0001	23.60	<0.0001	27.70	<0.0001	27.50	<0.0001	21.30	<0.0001	16.50	<0.0001
Accession	1.14	0.2250	1.82	0.0003	2.40	<0.0001	2.99	<0.0001	3.03	<0.0001	3.65	<0.0001	4.23	<0.0001	4.60	<0.0001	5.02	<0.0001
Accession^*^Block	1.40	0.2367	4.70	0.0302	2.90	0.0886	4.00	0.0455	5.50	0.0190	12.00	0.0005	7.10	0.0077	5.70	0.0170	3.00	0.0833
Control Col-0	30.99	<0.0001	149.27	<0.0001	50.24	<0.0001	21.42	<0.0001	14.83	<0.0001	0.28	0.5976	0.66	0.4180	0.10	0.7547	1.19	0.2761
Broad-sense heritability *H*^2^	0.16^ns^		0.61^***^		0.74^***^		0.79^***^		0.78^***^		0.81^***^		0.85^***^		0.86^***^		0.88^***^	
**B. 30°C–CUT**																		
**Traits**	**Symptoms D3**		**Symptoms D4**		**Symptoms D5**		**Symptoms D6**		**Symptoms D7**		**Symptoms D10**							
**Model terms**	***F*** **or LRT**	***P***	***F*** **or LRT**	***P***	***F*** **or LRT**	***P***	***F*** **or LRT**	***P***	***F*** **or LRT**	***P***	***F*** **or LRT**	***P***						
*Block*	0.00	1.0000	1.40	0.2367	2.50	0.1138	3.20	0.0736	1.60	0.2059	0.00	1.0000						
Accession	2.04	<0.0001	1.86	0.0002	1.93	0.0001	2.65	<0.0001	2.98	<0.0001	4.21	<0.0001						
Accession^*^Block	0.00	1.0000	3.10	0.0783	4.30	0.0381	0.90	0.3428	0.00	1.0000	0.50	0.4795						
Control Col-0	24.95	<0.0001	13.80	0.0006	11.59	0.0007	0.15	0.6950	0.02	0.8967	–	–						
Broad-sense heritability *H*^2^	0.71^***^		0.63^***^		0.63^***^		0.76^***^		0.82^***^		0.88^***^							

*F, F value resulting from the test of fixed effect; LRT, LRT value resulting from the likelihood ratio test*.

ns, non significant;

*** *P < 0.001; Italic terms indicate random effects*.

**Table 2:** Natural variation among natural accessions for disease index in the UNCUT condition.

**A. 27°C–UNCUT**																		
**Traits**	**Symptoms D3**		**Symptoms D4**		**Symptoms D5**		**Symptoms D6**		**Symptoms D7**		**Symptoms D8**		**Symptoms D9**		**Symptoms D11**		**Symptoms D13**	
**Model terms**	***F*** **or LRT**	***P***	***F*** **or LRT**	***P***	***F*** **or LRT**	***P***	***F*** **or LRT**	***P***	***F*** **or LRT**	***P***	***F*** **or LRT**	***P***	***F*** **or LRT**	***P***	***F*** **or LRT**	***P***	***F*** **or LRT**	***P***
*Block*	3.50	0.0614	18.70	<0.0001	44.40	<0.0001	72.30	<0.0001	63.70	<0.0001	75.30	<0.0001	58.40	<0.0001	19.60	<0.0001	19.40	<0.0001
Accession	1.65	<0.0001	2.62	<0.0001	3.68	<0.0001	5.22	<0.0001	5.87	<0.0001	6.84	<0.0001	6.53	<0.0001	5.30	<0.0001	5.26	<0.0001
Broad-sense heritability *H*^2^	0.37^***^		0.57^***^		0.71^***^		0.81^***^		0.83^***^		0.86^***^		0.85^***^		0.81^***^		0.81^***^	
**B. 30°C–UNCUT**																		
**Traits**	**Symptoms D3**		**Symptoms D4**		**Symptoms D5**		**Symptoms D6**		**Symptoms D7**		**Symptoms D10**							
**Model terms**	***F*** **or LRT**	***P***	***F*** **or LRT**	***P***	***F*** **or LRT**	***P***	***F*** **or LRT**	***P***	***F*** **or LRT**	***P***	***F*** **or LRT**	***P***						
*Block*	7.90	0.0049	11.60	0.0007	48.00	<0.0001	0.60	0.4386	0.00	1.0000	0.00	1.0000						
Accession	1.86	<0.0001	1.86	<0.0001	2.87	<0.0001	2.55	<0.0001	2.72	<0.0001	1.28	0.0357						
Control Col-0	1.08	0.2989	2.52	0.1136	0.01	0.9299	2.83	0.1582	0.10	0.7652	–	–						
Broad-sense heritability *H*^2^	0.44^***^		0.47^***^		0.67^***^		0.62^***^		0.65^***^		0.14^ns^							

*F, F value resulting from the test of fixed effect; LRT, LRT value resulting from the likelihood ratio test*.

ns, non significant;

*** *P < 0.001; Italic terms indicate random effects*.

**Figure 2:**
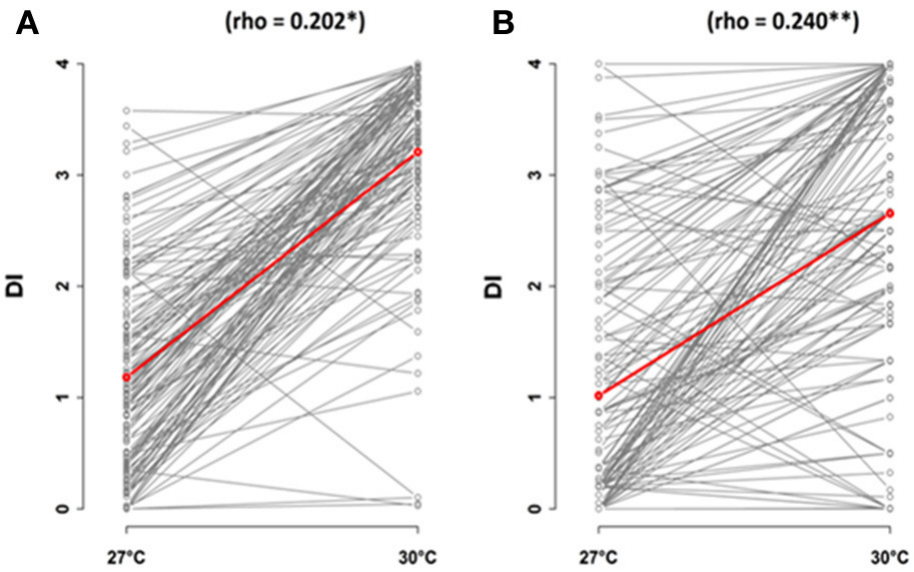
Correlations of temperature conditions, 27 and 30°C, in response to *R. solanacearum* inoculation of the worldwide collection of *A. thaliana*, at 5 days after inoculation. Inoculation was performed on 4-week-old plants for which roots have been cut **(A)** or left uncut **(B)**. The red line represents the mean of disease index. dai, days after inoculation; DI, disease index.

In both methods of inoculation, variation in disease index at 30°C was significantly correlated with variation in disease index at 27°C (Figure 2). However, the correlation coefficient of Pearson was strongly different from 1 (CUT condition, 95% confidence intervals: 0.037–0.357; UNCUT condition, 95% confidence intervals: 0.087–0.383). The observation of crossing reaction norms between 27 and 30°C suggests that the genetic architecture of the response of *A. thaliana* to the *R. solanacearum* GMI1000 strain is different between these two temperatures (Figure 2).

### A major QTL corresponding to the immune receptor pair *RPS4/RRS1* locus confers QDR to the *R. solanacearum* GMI1000 strain at 27°C

**Figure 3:**
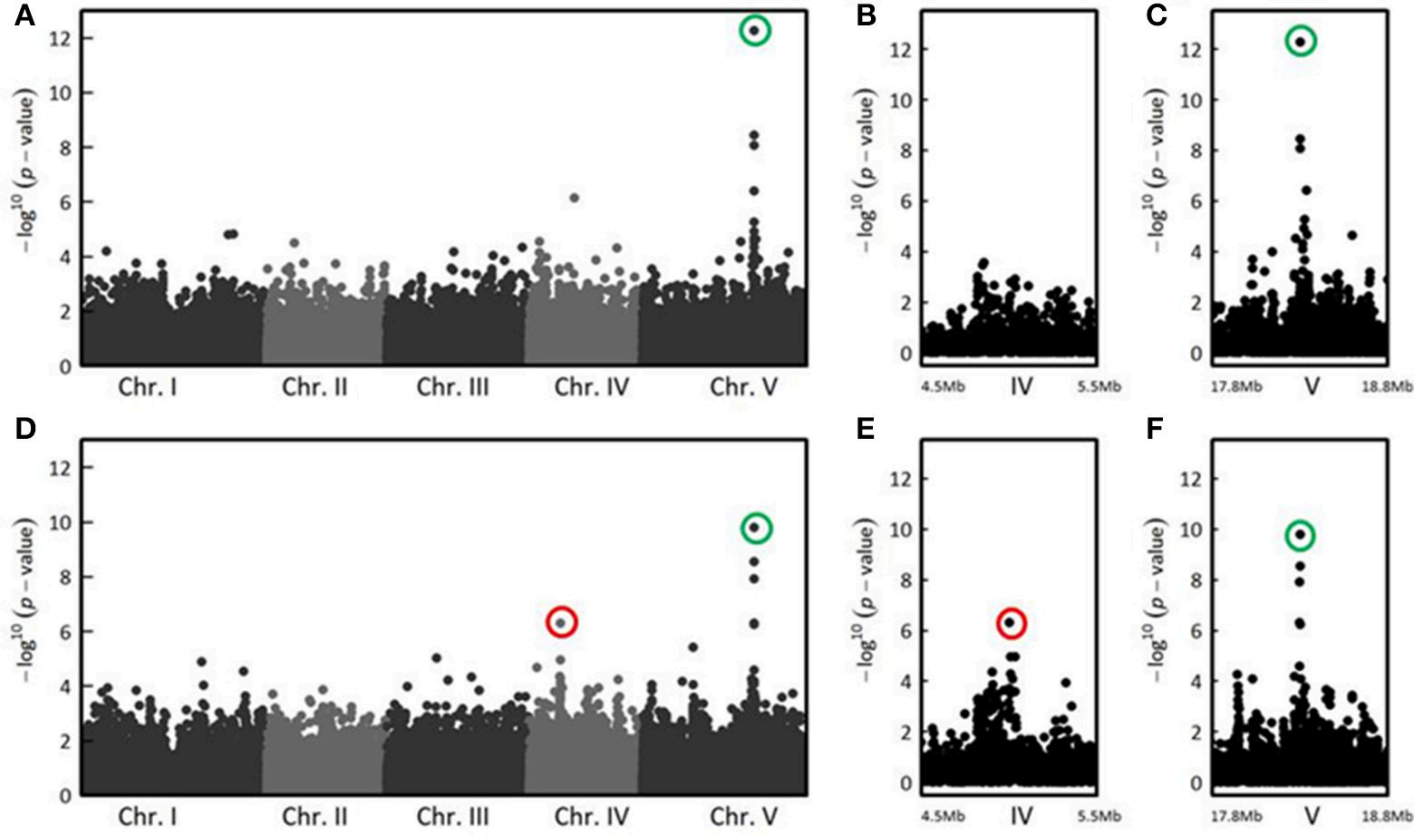
The genetics of quantitative disease resistance to *R. solanacearum* GMI1000 strain at 27°C, identified by GWA mapping at 13 dai in the CUT and UNCUT conditions of inoculation. Whole genome scan of 214,051 single-nucleotide polymorphisms (SNPs) for association with disease index at 13 dai across 152 accessions that have germinated for which roots have been cut **(A)**. Zoom showing the absence of QTLs of resistance in the CUT condition of inoculation on the chromosome IV, compared with the UNCUT condition thereafter **(B)**. Zoom spanning a genomic region on the chromosome V from 17.8 to 18.8 Mb containing the QTL of resistance located in the *RPS4/RRS1* locus **(C)**. Whole genome scan of 214,051 single-nucleotide polymorphisms (SNPs) for association with disease index at 13 dai across 163 accessions that have germinated for which roots remained uncut **(D)** and focus on two genomic regions corresponding to two QTLs of resistance observed on chromosomes IV **(D)** and chromosome V **(E)**. The red circle indicates the top SNP (SNP-4-5080256, *P* = 4.93 × 10^−7^, MARF = 0.429) corresponding to a QTL of resistance detected at the beginning of chromosome IV in the UNCUT condition. The green circles highlight the top SNPs corresponding to the major QTL of resistance detected on the long arm of chromosome V in the UNCUT (SNP-5-18325032, *P* = 5.37 × 10^−13^, MARF = 0.125) and CUT (SNP-5-18325565, *P* = 1.60 × 10^−10^, MARF = 0.252) conditions.

In the CUT condition, GWA mapping revealed a unique peak of association on the long arm of chromosome 5 whose significance increased with the stages of infection (Figure S1). Thirteen days post-inoculation, the three most associated SNPs (SNP-5-18325032, *P* = 5.37 × 10^−13^, MARF = 0.125; SNP-5-18325565, *P* = 3.55 × 10^−9^, MARF = 0.237; SNP-5-18325915, *P* = 8.65 × 10^−9^, MARF = 0.151) were located in the *RPS4* gene (*At5g45250*) (Figures 3A–C), that encodes the RPS4 immune receptor. Previously described as cooperating genetically and molecularly with RRS1-R for resistance to *R. solanacearum* (Narusaka et al., 2009), *RPS4* and *RRS1* gene are localized near each other and are inserted in opposite directions. Therefore, the identification of the *RPS4/RRS1* locus as the major resistance QTL to *R. solanacearum* confirms the suitability of a GWA mapping approach to investigate the genetic bases of resistance responses to this bacterial pathogen.

In the UNCUT condition, we detected two neat peaks of association. The first association peak, at the beginning of chromosome IV, was detected at the late stages of infection (Figure S2), with the top SNP (SNP-4-5080256, *P* = 4.93 × 10^−7^, MARF = 0.429) located in a transposable element gene (*At4g08100*) belonging to the gypsy-like retrotransposon family (Figures 3D,E). The significance of the second association peak located on the chromosome V also increased with the stages of infection (Figure S2). Similarly to the CUT condition, the four most associated SNPs at 13 dai (SNP-5-18325565, *P* = 1.60 × 10^−10^, MARF = 0.252; SNP-5-18325032, *P* = 2.80 × 10^−9^, MARF = 0.123; SNP-5-18322558, *P* = 1.19 × 10^−8^, MARF = 0.301; SNP-5-18323844, *P* = 4.85 × 10^−7^, MARF = 0.295) were also found in *RPS4* (Figures 3D,F). These results suggest that although the major QTL was common for the two methods of inoculation, medium/minor QTLs can be specific to the inoculation method and/or to the stage of infection.

### Different genetic architectures at 30°C highlight different plant defense response mechanisms

**Figure 4:**
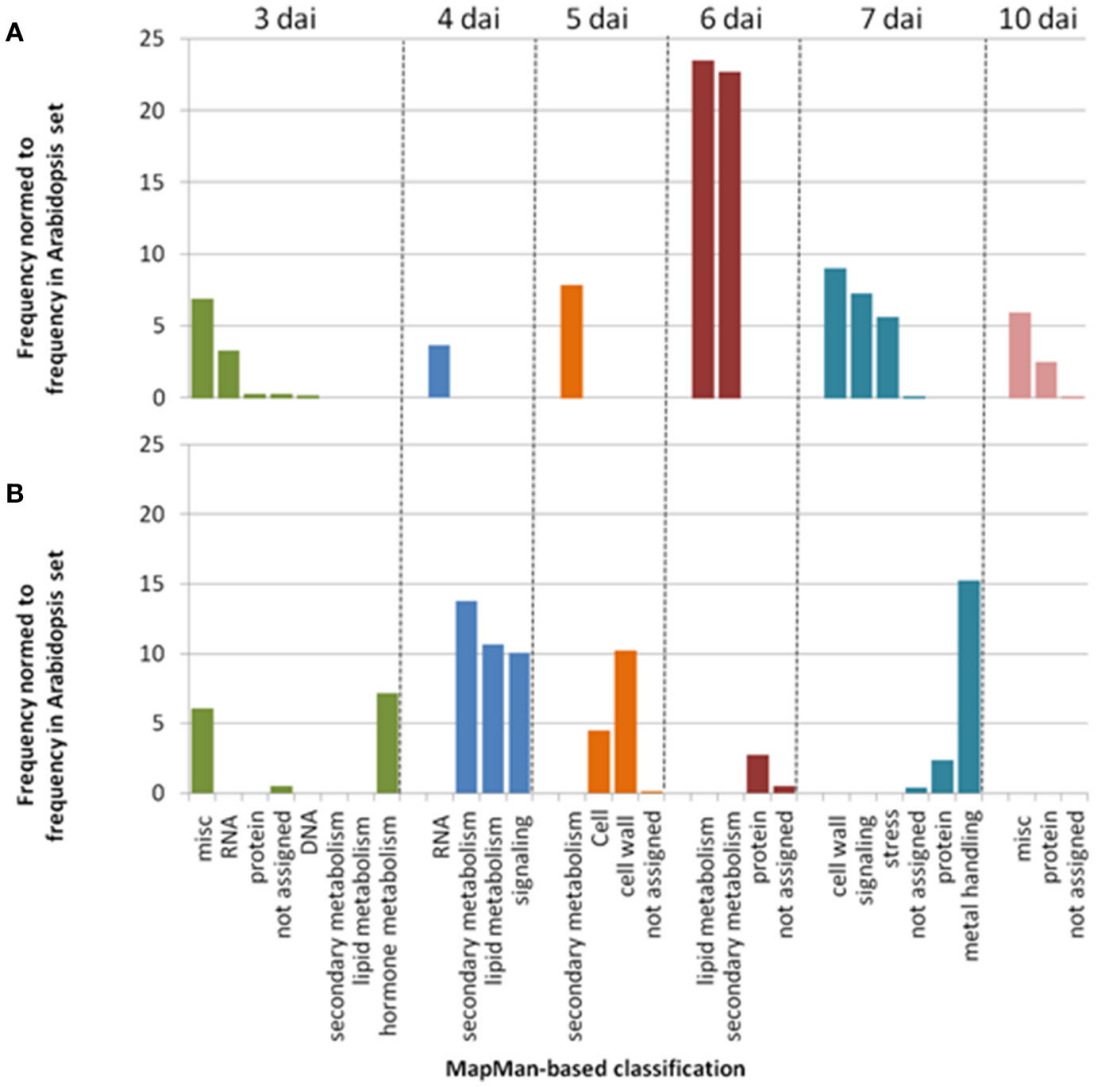
Simplified representation of MapMan classification pathways found to be significantly over-represented (*P* < 0.01). Genes list were retrieved from enrichment tests performed using the SNPs in the 0.1% upper tail of the −log10 *p*-value distribution for each time point of phenotyping in the CUT **(A)** and UNCUT **(B)** conditions at 30°C (Tables S6, S7).

In agreement with the small correlation coefficients observed at the phenotypic level between 27 and 30°C (Figure 2), GWA mapping revealed a different genetic architecture at 30°C, with no association peak located in the *RPS4/RRS1* locus or at its close vicinity (Figures S3, S4). In addition, for each phenotyping time point, less than 2% of the 100 most significant associated SNPs (i.e., top SNPs) were shared between the two inoculation methods at 30°C (Figures S3, S4), suggesting that the genetic basis of response to *R. solanacearum* largely differs between the CUT and UNCUT conditions. Different significantly over-represented functional classes assigned using the MapMan classification, (supported from >97.5% bootstrap replicate), in which gene lists corresponding to the top SNPs for each “CUT/UNCUT condition × time point of phenotyping” fall in, also support this observation. In the CUT condition, numerous genes involved in RNA processing and RNA regulation were found at 3 and 4 dai, whereas genes related to the perception and the regulation of plant response to biotic or abiotic stresses (i.e., leucine rich repeat protein, NBS-LRR protein, Cystein rich receptor like kinase protein, cytochrome P450) and protein degradation were mostly found at later stages of infection (Table S6, Figure 4A). By contrast, genes retrieved in the UNCUT condition were significantly over-represented in metabolic pathways at 3–5 dai, with genes having a role in hormone metabolism, defense response to biotic and abiotic stresses (CYP450 family 705 subfamily A; flavonoid synthase), cell wall modification and signaling. Genes retrieved at 6 and 7 dai reflect an intense protein metabolism activity with numerous genes involved in protein synthesis and degradation (Table S7, Figure 4B).

**Figure 5:**
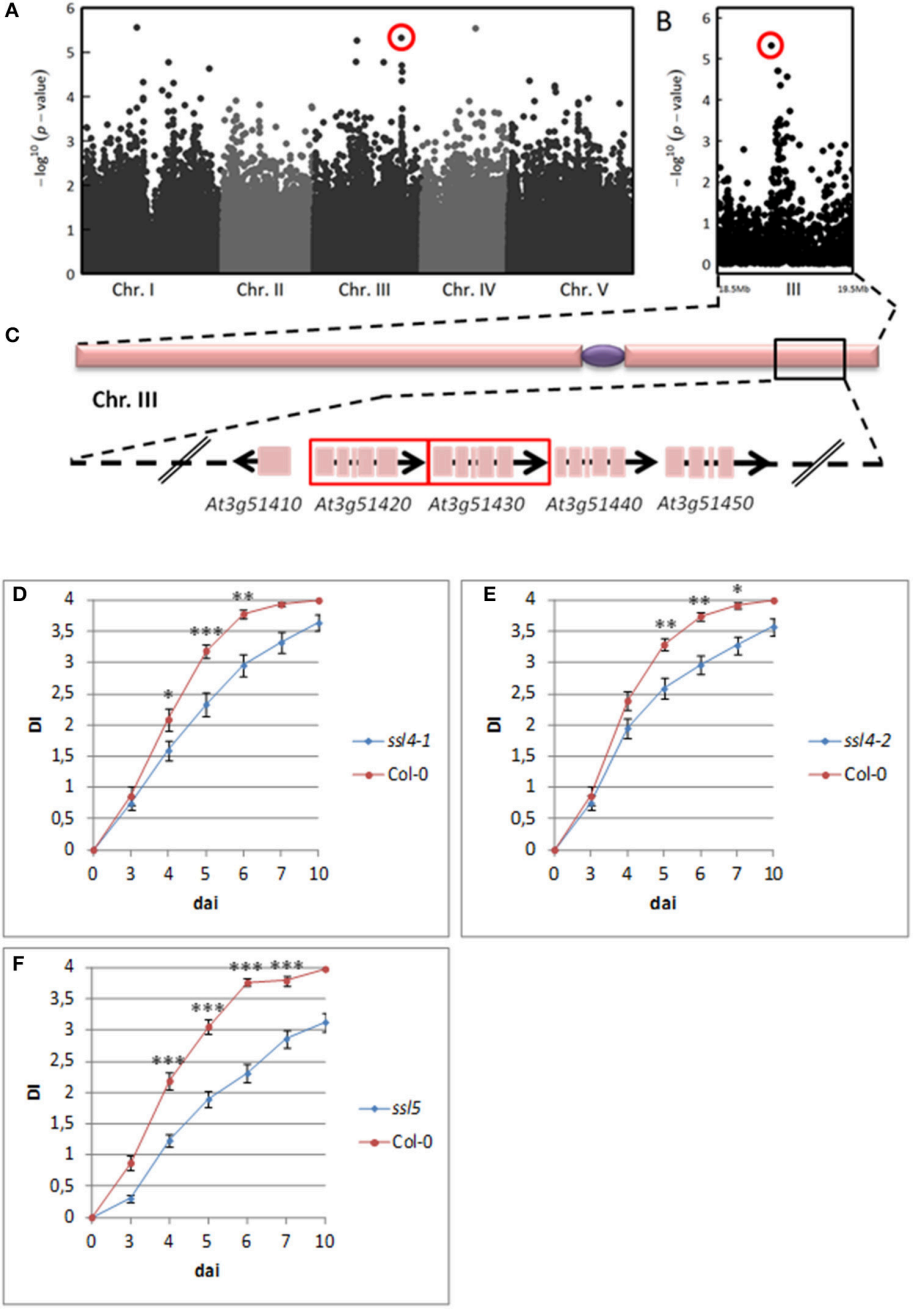
The genetics of quantitative disease resistance to *R. solanacearum* GMI1000 strain at 30°C, identified by GWA mapping at 5 dai in the CUT condition of inoculation. Whole genome scan of 214,051 single-nucleotide polymorphisms (SNPs) for association with disease index at 5 dai across 141 accessions having germinated **(A)**. Zoom spanning a genomic region on the chromosome III from 18.5 to 19.5 Mb containing a QTL of early plant defense response to *R. solanacearum* **(B)**. Zoom showing the 80 Kb genomic region underlying the QTL of early plant defense response to *R. solanacearum*, containing 31 annotated genes. Red frames indicate genes coding for *AtSSL4* (*At3g51420*) and *AtSSL5* (*At3g51430*) **(C)**. Dynamics of disease symptoms after inoculation with the GMI1000 strain in wild type Col-0 genetic background and *ssl4-1* mutant at 30°C. Means ± *SD* of the means (*ssl4-1 n* = 60; Col-0 *n* = 40) from three independent inoculations **(D)**. Dynamics of disease symptoms after inoculation with the GMI1000 strain in wild type Col-0 genetic background and *ssl4-2* mutant at 30°C. Means ± SD of the means (*ssl4-2 n* = 83; Col-0 *n* = 59) from four independent inoculations **(E)**. Dynamics of disease symptoms after inoculation with the *R. solanacearum* GMI1000 strain in wild type Col-0 genetic background and *ssl5* mutant at 30°C. Means ± SD of the means (*ssl5 n* = 124; Col-0 *n* = 77) from six independent inoculations **(F)**. Symbols ^*^, ^**^, and ^***^ denote significant difference observed between Col-0 and each mutant at *P* < 0.05, *P* < 0.01, and *P* < 0.001 respectively, in Kruskal-Wallis analyses. The red circle indicates the top SNP (SNP-3-18972992, *P* = 4.70 × 10^−6^, MARF = 0.142) corresponding to the major QTL of resistance detected at the end of chromosome III.

**Figure 6:**
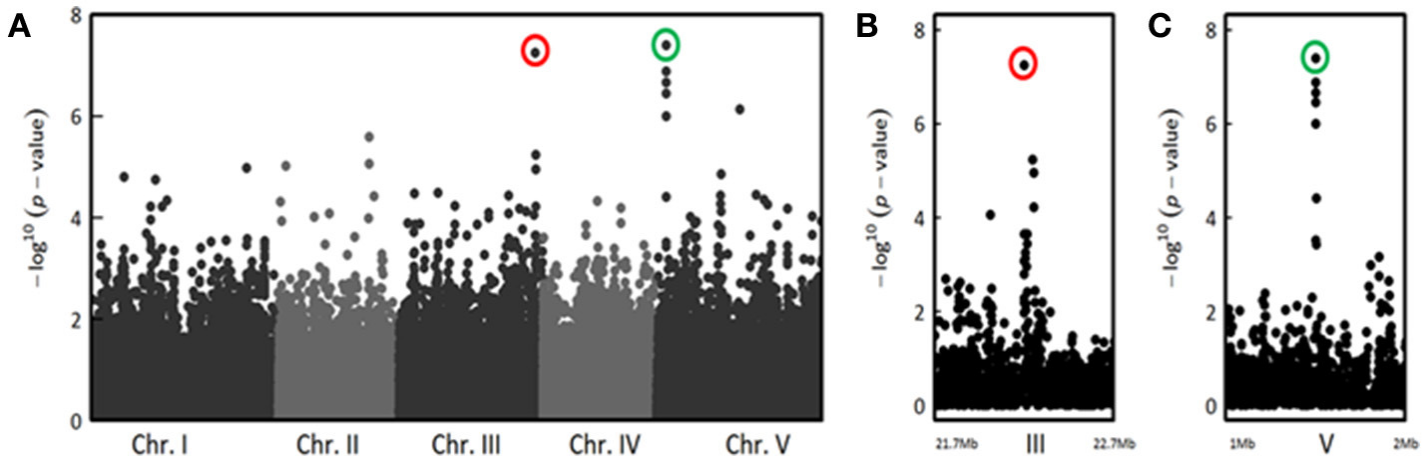
The genetics of quantitative disease resistance to *R. solanacearum* GMI1000 strain at 30°C, identified by GWA mapping at 6 dai in the UNCUT condition of inoculation. Whole genome scan of 214,051 single-nucleotide polymorphisms (SNPs) for association with disease index at 6 dai across 158 accessions that have germinated **(A)** and focus on two genomic regions corresponding to QTLs of early plant defense to *R. solanacearum* observed at the end of chromosome III **(B)** and at the beginning of chromosome V **(C)**. The red circle indicates the top SNP (SNP-3-22260125, *P* = 5.65 × 10^−8^, MARF = 0.127) corresponding to a QTL of resistance detected at the end of chromosome III. The green circle highlights the top SNP (SNP-5-1530992, *P* = 4.04 × 10^−8^, MARF = 0.189) corresponding to a QTL of resistance detected at the beginning of chromosome V.

Interestingly, for both inoculation methods, we observed playful dynamics of the association peaks along the infection stages (Figures S3, S4). For example, in the CUT condition, we detected multiple association peaks specific to the 5 dai stage (Figure 5A). In particular, GWA mapping revealed an association peak located at the end of chromosome III supported by 12 SNPs with an association score above 3, embedded in a region of 80 kb (Figure 5B). Similarly, in the UNCUT condition, GWA mapping revealed multiple association peaks specific to the 6 dai stage (Figure 6A). More specifically, two association peaks caught our attention as they are supported by numerous top SNPs, including those the most significantly associated to the plant response to *R. solanacearum* at the 6 dai stage (Table S3), and overlap with candidate genes known to be involved in plant response to different pathogens. The first one is located at the end of chromosome III, with the top SNP (SNP-3-22-260-125) located in the promoter region of the *EIF4G* (*At3g60240*) gene (Figure 6B). The second one is located at the beginning of chromosome V, with the six most associated SNPs all located within the *CesA3* (*At5g05170*) gene (Figure 6C).

### QDR to the *R. solanacearum* GMI1000 strain at 30°C in the cut condition is conferred by a gene encoding for a strictosidine synthase-like protein 4

Since neat association peaks at the early stages of infection could be detected (Figures 5B, 6B,C), we therefore investigated the molecular mechanisms underlying plant response to *R. solanacearum* at 30°C. To functionally validate one of those peaks, we focused on the QTL region identified at the bottom of chromosome III at 30°C in the CUT condition (Figure 5B). We screened this 80-kb QTL region (Figure 5C) by genotyping 38 T-DNA insertion mutants publically available and corresponding to 21 candidate genes (Table S2). T-DNA insertions were confirmed by genotyping and sequencing the T-DNA flanking sequences for 24 mutants. Homozygous progenies were produced and their responses to *R. solanacearum* at 30°C were scored. Out of the 21 mutants tested, two allelic mutants of the *At3g51420* gene, named *ssl4-1* (N683907), and *ssl4-2* (N684981), for which the altered gene expression was confirmed by RT-qPCR (Figure S7), exhibited a significant higher level of resistance from 4 to 7 dai compared to wild-type Col-0 (Figures 5D,E). These data indicate that *At3g51420* is a gene that increases the susceptibility to the *R. solanacearum* GMI1000 strain at 30°C. This gene, that encodes for a strictosidine synthase-like protein 4 (SSL4), belongs to a family of four genes *(SSL4, 5, 6*, and *7)* organized in tandem at this locus. Interestingly, knocking-down of one of the three other genes of the SSL family [*ssl5* mutant (N659319)], has also led to a higher level of resistance from 4 to 7 dai (Figures 5C,F). Representative symptoms observed at 5 dai for *ssl4-1, ssl4-2*, and *ssl5* mutants compared to Col-0 accession are shown in Figure S8. Together, our data reveal that both *SSL4* and *SSL5* can be considered as susceptibility genes since they promote the development of wilting symptoms of Col-0 plants in response to the GMI1000 strain.

## Discussion

In this study, we explored the genetic diversity of responses to *R. solanacearum* in *A. thaliana* under elevated temperature conditions (i) to estimate the extent of genetic diversity in response to this combined abiotic-biotic stress, (ii) to describe the genetic architecture of plant response to the bacterial wilt disease, and (iii) to unravel the genetic bases of QDR mechanisms that are efficient under a permanent increase of 3°C. Because *R. solanacearum* is a soil borne pathogen that penetrates into the plants through the roots, we also aimed at studying how the infection process could modulate the response of *A. thaliana* to *R. solanacearum*. For this, two inoculation methods were used: (i) the CUT condition in which the roots were cut allowing the bacteria to access directly to the xylem vessels and (ii) the UNCUT condition, in which the roots were not injured to mimic natural infection.

### Significant natural genetic variation is observed in *A. thaliana* challenged with *R. solanacearum*, at both 27 and 30°C

In our study, we observed a drastic effect of elevated temperature on *A. thaliana* defense response to *R. solanacearum*. At 30°C, most accessions were on average more susceptible, with wilting disease progression being always faster regardless of the inoculation condition used. These results corroborate several previous studies describing that elevated temperatures generally inhibit resistance responses (Whitham et al., 1996; Hwang et al., 2000; de Jong et al., 2002; Xiao et al., 2003; Wang et al., 2009; Zhu et al., 2010; Menna et al., 2015). At the same time, we also observed an extensive genetic diversity of response among *A. thaliana* accessions challenged with the GMI1000 strain as well as high estimates of broad-sense heritability across the stages of infection at 30°C, as it was also the case at 27°C. These results reveal the existence of resistant accessions to *R. solanacearum* with a genetically controlled response at the two temperatures tested. However, the crossing reaction norms observed between 27 and 30°C suggest that the genetic architecture of *A. thaliana* for the response to *R. solanacearum* can largely depend on abiotic conditions. Such a temperature-dependent genetic architecture has been previously reported in *A. thaliana* for diverse phenotypic traits such as flowering time (Lempe et al., 2005) and fertility-related traits (Bac-Molenaar et al., 2015; Thoen et al., 2017).

### The major QTL associated with resistance to *R. solanacearum* at 27°C, underlying the *RPS4/RRS1* locus, is not detected at 30°C

Whatever was the method of inoculation, GWA mapping revealed a major QTL on the long arm of chromosome V with an increasing temporal significance at 27°C, suggesting that a simple genetic architecture governs the defense response to the *R. solanacearum* GMI1000 strain. For both inoculation conditions, the most significant SNPs were found in the *RPS4/RRS1* locus, known to be involved in one of the best characterized resistance mechanism to *R. solanacearum* in *A. thaliana* (Deslandes et al., 2002; Le Roux et al., 2015). Interestingly, a previous GWA study also identified this locus to be responsible for specific QDR in response to the *Xcc* CFBP6943 strain (race 6) (Debieu et al., 2016). Therefore, our results demonstrate the potential of GWAS to identify major loci associated with natural variation of resistance to *R. solanacearum*, as previously shown for several other bacterial pathogens, predominantly in *A. thaliana* (Huard-Chauveau et al., 2013; Debieu et al., 2016; Roux and Bergelson, 2016; Bartoli and Roux, 2017).

By contrast, at all phenotyping time points, no significant association peak underlying the *RPS4/RRS1* genomic region was detected at 30°C. This result supports, for the first time, that the resistance mechanism conferred by this immunoreceptor pair is impaired at 30°C. The thermosensitivity of PTI and *R*-gene-mediated defense for different pathogens in different plant species has been reported in several studies (Whitham et al., 1996; Hwang et al., 2000; de Jong et al., 2002; Xiao et al., 2003; Yang and Hua, 2004; Wang et al., 2009; Menna et al., 2015). In addition, the dwarf phenotype of several autoimmune mutants such as *bon1* and *snc4-1D* or *snc2-1D, mkk1 mkk2*, and *bir1-1* can be totally or partially suppressed above 28°C (van Wersch et al., 2016). This is also the case for the autoimmune response of *A. thaliana* transgenic lines constitutively expressing the *RPS4* immune receptor (Heidrich et al., 2013). To date, the mechanisms involved in such temperature-inhibition of immune responses remain largely uncharacterized. Interestingly, Cheng et al. (2013) proposed that plants preferably activate ETI signaling at low temperatures while they use PTI signaling at elevated temperatures.

In this first GWA study on *R. solanacearum*, we have identified at 27°C, in both inoculation methods, the *RRS1-*containing locus, originally mapped by a conventional positional cloning approach (Deslandes et al., 2002). Therefore, we clearly showed that the study of natural diversity of plant response to *R. solanacearum* is a powerful strategy to fine map genomic regions governing susceptible and resistance responses to bacterial wilt. We also showed that an increase in temperature strongly impacts plant defense responses to *R. solanacearum* preventing the detection of *RSP4/RRS1* locus at 30°C most likely due to a loss of function of this immunoreceptor pair.

### GWA analyses at 30°C highlight playful dynamics in the genetics of response to *R. solanacearum* depending on the inoculation procedure

Owing to GWA analyses carried out on the phenotypic data monitored every day after inoculation, a more pronounced playful dynamics of association peaks along the infection stages was observed at 30°C than at 27°C. Among the multiple peaks identified, comparison of the 100 top SNPs at each phenotyping time point revealed that less than 2% were shared between the CUT and UNCUT conditions, suggesting that the genetic basis of response to *R. solanacearum* largely depend on the infection process. Enrichment tests performed to determine the most important processes set up by the plant in response to *R. solanacearum* at 30°C for both inoculation conditions also support these specificities. Indeed, only few genes were found to be significantly over-represented in both conditions and correspond to the same miscellaneous functional class at 3 dai. Interestingly, they mainly encode for CYP450 subfamily 71 and 705 (CYP705) proteins as well as uridine diphosphate glycosyltransferases (UGT) known to be involved in plant response to biotic and abiotic stresses (Ross et al., 2001; Bak et al., 2011). In the CUT condition, at early infection stages, most genes underlying significantly over-represented biological processes correspond to regulatory genes. For instance, many genes encoding transcription factors (TF) were retrieved at 3 and 4 dai, suggesting a rapid transcriptional reprogramming in the early stages of infection. Interestingly, at later infection stages (i.e., from 5 to 7 dai), many genes are involved in calcium-dependent signaling, pathogen perception (TIR-NBS-LRR proteins) and regulation of biotic and abiotic stress responses (LRR- and cystein rich receptor like kinase proteins). For example, CRK28 and its interacting partner CRK29, were recently demonstrated to enhance plant immune responses (Yadeta et al., 2017). By contrast, genes retrieved with the UNCUT condition are predominantly involved in metabolic processes. In particular, genes underlying significantly over-represented biological processes at early infection stages correspond to Brassicaceae-specific CYP705 family and flavonol synthases. Even if the role of the CYP705 family is poorly characterized, the CYP705A1 was described as participating in the formation of a volatile homoterpene, (E)-4,8-dimethyl-1,3,7-nonatriene (DNMT), involved in the *A. thaliana* resistance to the root-rot pathogen *Pythium irregular* (Sohrabi et al., 2015). In addition, flavonoids are well-described molecules known to be important for response to other organisms or environmental stresses (Mierziak et al., 2014). At later infection stages (i.e., from 5 to 6 dai), candidate genes have primarily a role in cell wall formation. Interestingly, among virulence factors used by *R. solanacearum*, cell wall-degrading enzymes such as pectinolytic and cellulytic enzymes, can promote invasion of roots and/or penetration of xylem vessels (Liu et al., 2005). Such specific processes could allow certain accessions to restrict bacterial penetration at the early stages of infection on intact roots. Indeed, through the inoculation of plants with intact or cut roots, Turner et al. (2009) dissected in *R. solanacearum* the steps involved in root colonization of the model legume plant *Medicago truncatula*. While two distinct T3Es (Gala7 and AvrA) are involved in the early stages of colonization, suggesting that specific basal defense mechanisms are rapidly manipulated by the bacteria, Gala7 alone was shown to play a major role in the later stages of infection in cut roots (Turner et al., 2009).

In addition to the association peak identified at 5 dai in the CUT condition, GWA analyses revealed two other neat association peaks in the UNCUT condition, with the top SNPs located in the promoter region of the *EIF4G* gene and within the *CesA3* gene. EIF4G is a translation initiation factor that belongs to protein families participating in the eukaryotic translation initiation complex. *EIF4G* was identified as a recessive resistance gene impairing multiplication of the cucumber mosaic virus (CMV) and of the turnip crinkle virus (TCV) in *A. thaliana* (Yoshii et al., 2004), or conferring high resistance to the rice yellow mottle virus in rice (Albar et al., 2006). The *CesA3* gene belongs to a family of 10 *CesA* genes identified in *A. thaliana* of which 9 encode cellulose synthase subunits with known function. CesA1, -2, -3(IXR1), -5, and -6(IXR2) were shown to participate in the cellulose production during the primary cell wall formation, while CesA4 (IRX5), -7 (IRX3), -8(IRX1), and -9 are involved in the secondary cell wall cellulose synthase complex (Endler and Persson, 2011). Hernández-Blanco et al. (2007) demonstrated that mutation in *CesA4, -7*, and *-8*, involved in secondary cell wall formation, enhanced tolerance to *Botrytis cinerea* and *Plectosphaerella cucumerina* fungi as well as to *R. solanacearum*. Strikingly, performing inoculation at 27°C with plants for which roots were cut, the authors showed that the *ixr1-1/cev1* mutant, altered in *CesA3* expression, was susceptible to *R. solanacearum*. Clearly, further studies are needed to functionally validate the implication of these candidate genes in tolerance to *R. solanacearum* at elevated temperatures in the early infection stages.

As in many cases in which genetic mapping approaches were developed to identify resistance mechanisms to pathogens (Cook et al., 2012; Roux et al., 2014; Fukuoka et al., 2015), we found in our study that several QTLs control QDR to *R. solanacearum* at 30°C in a playful manner specific to the inoculation method. Interestingly, the CUT condition highlights different processes that could be related to the perception and regulation pathways, dependent on temperature, of well-conserved microbial signatures such as ubiquitous effectors and PAMPs. On the other hand, the UNCUT condition revealed processes associated to plant development and metabolism which could reflect strategies set up by the plant to limit bacteria penetration and propagation in root tissues at the early stages of infection. As demonstrated by Turner et al. (2009) on the bacterial side and proposed by Roux et al. (2014), these processes could involve host components, sequentially manipulated by effectors during host colonization.

### A QDR to *R. solanacearum* efficient at 30°C is conferred by a strictosidine synthase-like protein 4

At 5 and 6 dai in the CUT condition, a neat association peak was identified at the end of the chromosome III, corresponding to another QTL involved in early defense response at 30°C. The phenotyping of several T-DNA insertion mutants corresponding to genes underlying this QTL allowed the functional validation of *At3g51420* (*AtSSL4*) as a gene of susceptibility to the *R. solanacearum* GMI1000 strain. Indeed, two allelic mutants were found to be significantly more resistant compared to wild-type Col-0 susceptible plants. The *At3g51420* gene belongs to a subset of four genes (*AtSSL4-AtSSL7*), arranged in tandem, which shows a strong similarity with genes encoding for hemomucin membrane-anchored immune proteins from *Drosophila melanogaster*. These proteins contain strictosidine synthase-like (SSL) domain related to plant and *Caenorhabditis elegans* SSL proteins. Because hemomucin proteins from *Drosophila* have immune-related functions, Sohani et al. (2009) explored the possible role of AtSSL4-AtSSL7 in plant defense response. Contrary to other *AtSSLs* genes, *AtSSL4* appeared to be slightly up-regulated upon wounding stress but not induced following inoculation with CMV and *Alternaria brassicicola* (Sohani et al., 2009). Still, these data do not exclude the possibility that *AtSSL4* could be regulated by other pathogens, like *R. solanacearum*. Among the knockout mutants corresponding to the three other *AtSSLs* genes, the *ssl5* mutant was also found to be more resistant to *R. solanacearum* at 30°C, from 3 to 10 dai. This result also suggests that *AtSSL5* could be a gene of susceptibility to *R. solanacearum*. Interestingly, unlike *AtSSL4, AtSSL5* expression is induced by salicylic acid, known to be a defense signaling compound, and is strongly up-regulated upon CMV and *A. brassicicola* inoculation (Sohani et al., 2009). In tomato, *SSL* gene expression is also up-regulated following the roots infection by *Fusarium oxysporum* f.sp. *radicis-lycopersici* (Manzo et al., 2016). Strictosidine synthase participates in the production of a wide range of monoterpenoid indole alkaloids important for plant defense response (Kibble et al., 2009) and Manzo et al. (2016) proposed that SSL could be involved in the incompatible interaction with the fungus. Thus, our result is the first demonstration of a role of plant SSLs in the resistance response to a bacterial pathogen. The enzymatic activity of proteins encoded by *AtSSL4-AtSSL7* has not been demonstrated yet (Kibble et al., 2009). Nonetheless, knowing that *SSLs* expression is highly regulated in different plant species upon pathogen attack, the resistance spectrum conferred by these mutants to other RSSC strains and other pathogens will be investigated at elevated temperatures.

## Conclusion

To our knowledge, this study is the first report of significant genetic variation in *A. thaliana* in response to *R. solanacearum* at elevated temperature. Our results also highlight that plant defense responses are strongly impacted at 30°C and differ according to the inoculation procedure. By combining a GWA mapping approach with the response to combined stresses, we identified three neat association peaks underlying QDR to *R. solanacearum* that are efficient at elevated temperature. Among the most interesting genes underlying the QTLs identified, we functionally validated *AtSSL4* as a susceptibility gene that plays a role in *R. solanacearum* infection at 30°C. Apart from *AtSSL4*, other genes underlying additional QTLs also constitute good candidates for QDRs that might represent new sources of sustainable resistance to the RSSC in the context of climate warming. In addition, the inhibition of the *RPS4/RRS1* resistance signaling at 30°C represents an interesting tool to investigate the impact of elevated temperature on ETI-related processes.

## Author contributions

NA, FV, FR, and RB conceived the experiments. NA, LT, FL, FV, and RB performed the experiments. NA and FR performed GWA analyses. NA, FV, and RB analyzed the other data. NA, FR, LD, FV, and RB wrote and corrected the manuscript. All authors approved the manuscript.

### Conflict of interest statement

The authors declare that the research was conducted in the absence of any commercial or financial relationships that could be construed as a potential conflict of interest.
