# Supplementary material for: Quantitative Disease Resistance under Elevated Temperature: Genetic Basis of New Resistance Mechanisms to Ralstonia solanacearum
Source: Front Plant Sci. 2017 Aug 22;8:1387. doi: 10.3389/fpls.2017.01387 (PMC5572249; doi:10.3389/fpls.2017.01387)
Supplement: Supplementary file 9 [file Presentation1.PPT]

## Slide 1
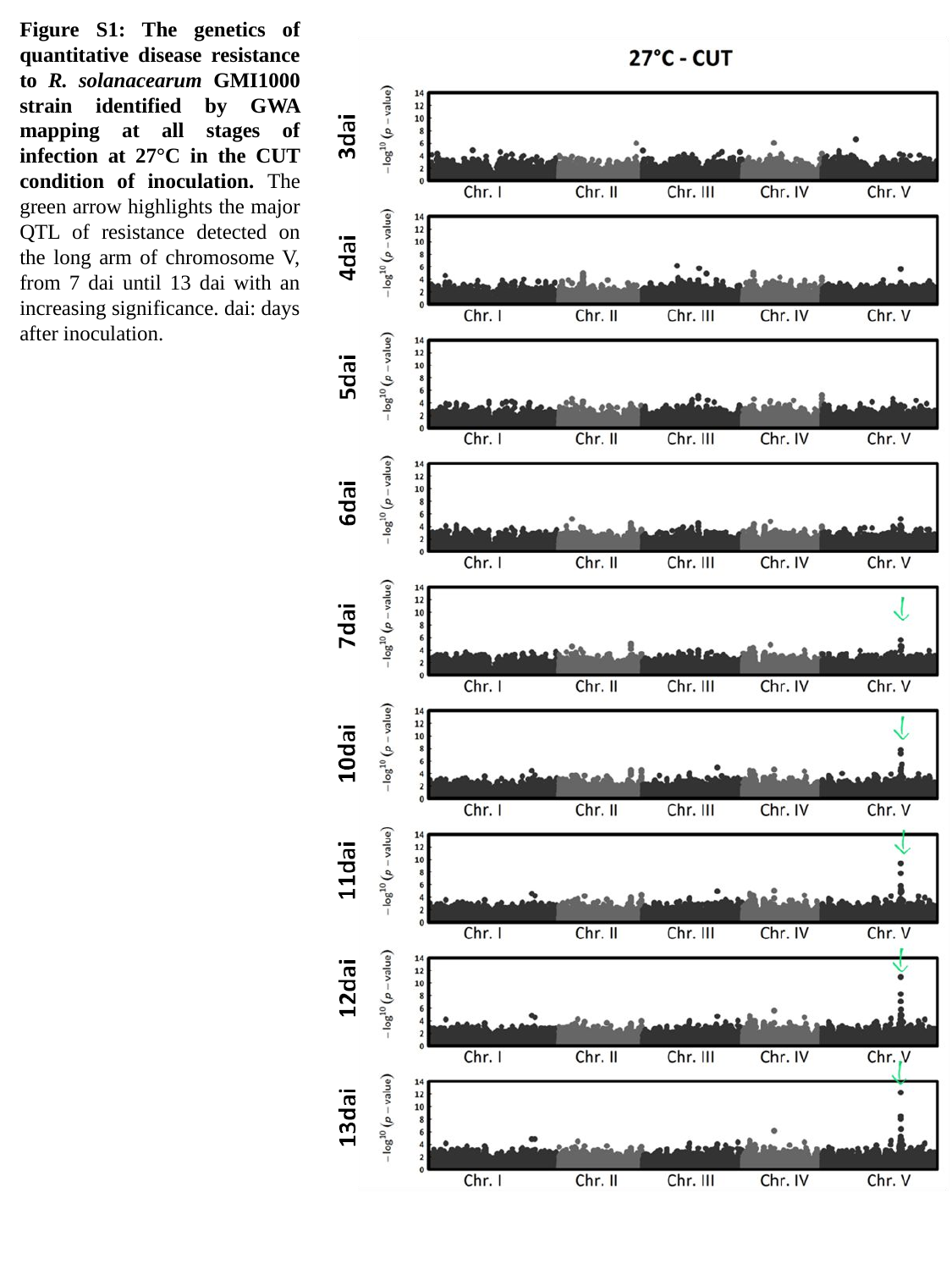

Figure S1: The genetics of quantitative disease resistance to R. solanacearum GMI1000 strain identified by GWA mapping at all stages of infection at 27°C in the CUT condition of inoculation. The green arrow highlights the major QTL of resistance detected on the long arm of chromosome V, from 7 dai until 13 dai with an increasing significance. dai: days after inoculation.

## Slide 2
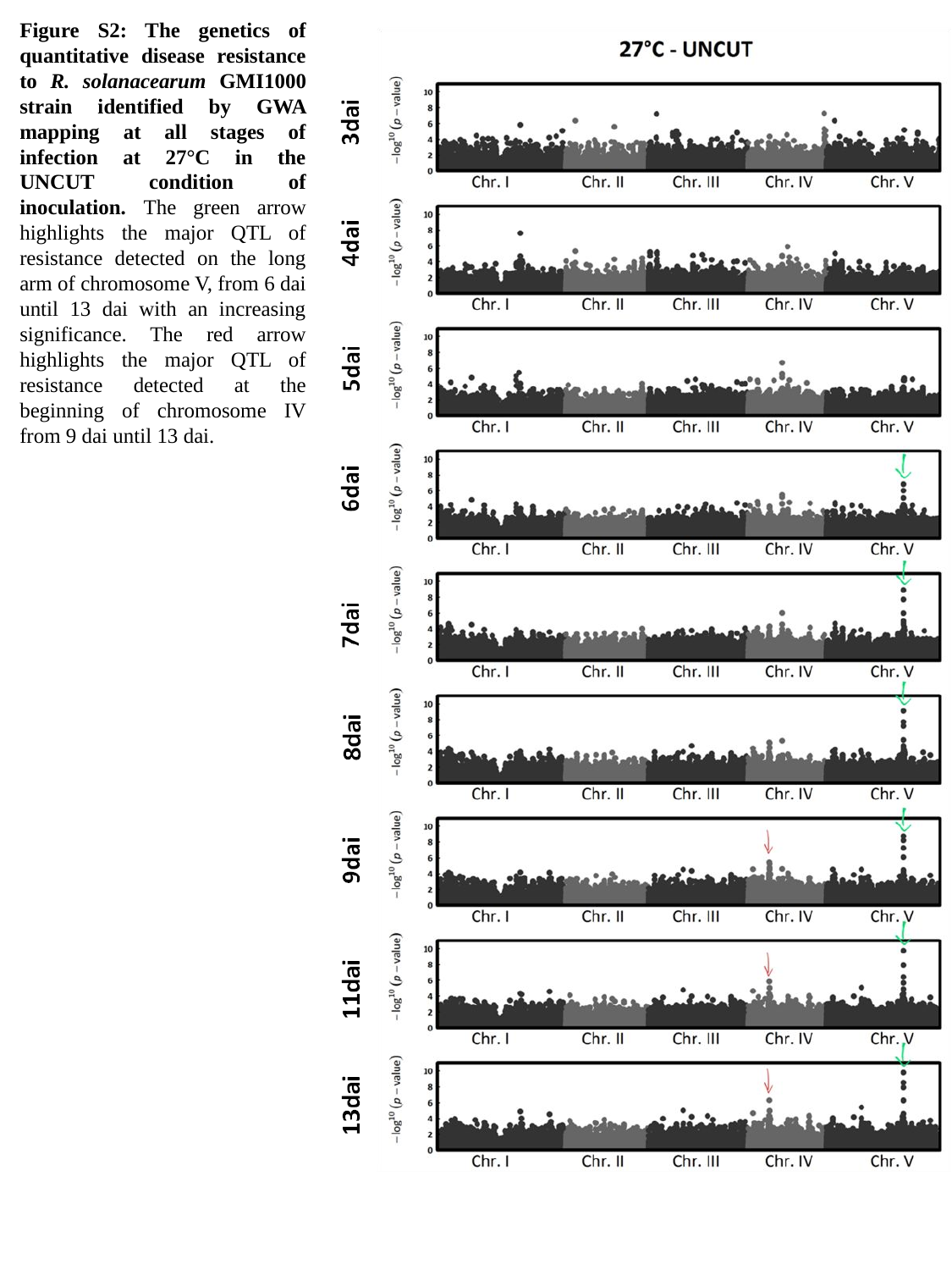

Figure S2: The genetics of quantitative disease resistance to R. solanacearum GMI1000 strain identified by GWA mapping at all stages of infection at 27°C in the UNCUT condition of inoculation. The green arrow highlights the major QTL of resistance detected on the long arm of chromosome V, from 6 dai until 13 dai with an increasing significance. The red arrow highlights the major QTL of resistance detected at the beginning of chromosome IV from 9 dai until 13 dai.

## Slide 3
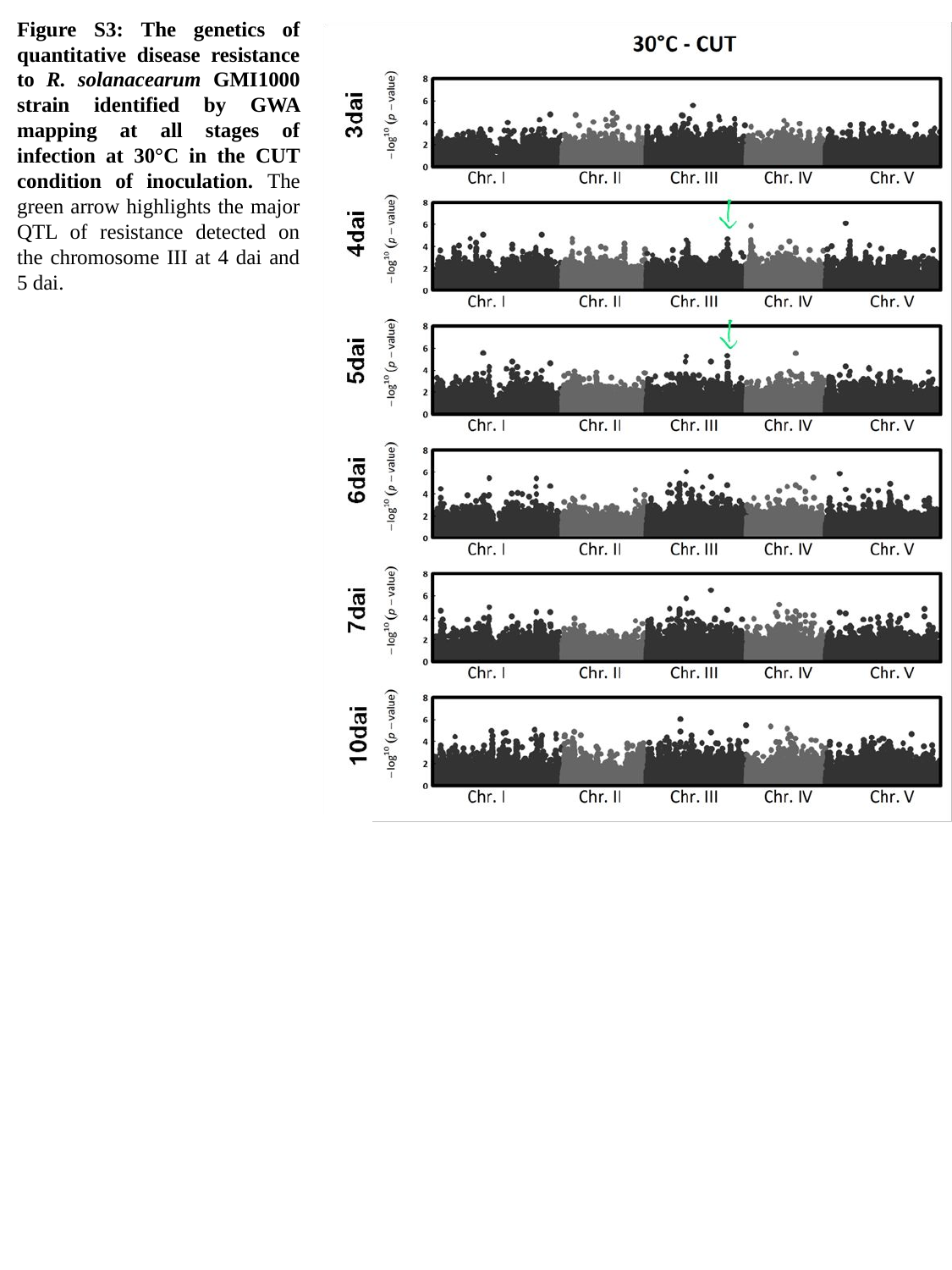

Figure S3: The genetics of quantitative disease resistance to R. solanacearum GMI1000 strain identified by GWA mapping at all stages of infection at 30°C in the CUT condition of inoculation. The green arrow highlights the major QTL of resistance detected on the chromosome III at 4 dai and 5 dai.

## Slide 4
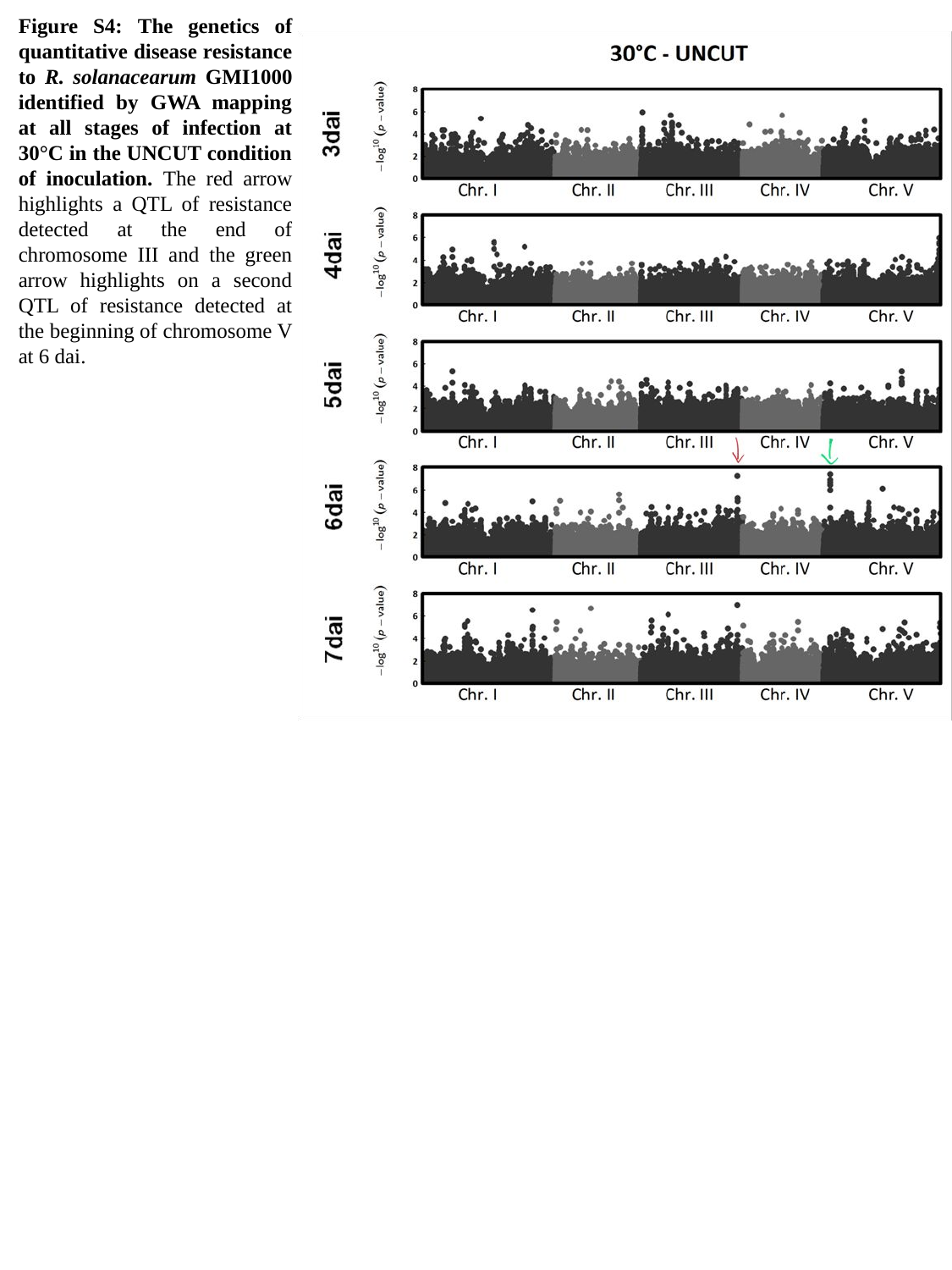

Figure S4: The genetics of quantitative disease resistance to R. solanacearum GMI1000 identified by GWA mapping at all stages of infection at 30°C in the UNCUT condition of inoculation. The red arrow highlights a QTL of resistance detected at the end of chromosome III and the green arrow highlights on a second QTL of resistance detected at the beginning of chromosome V at 6 dai.

## Slide 5
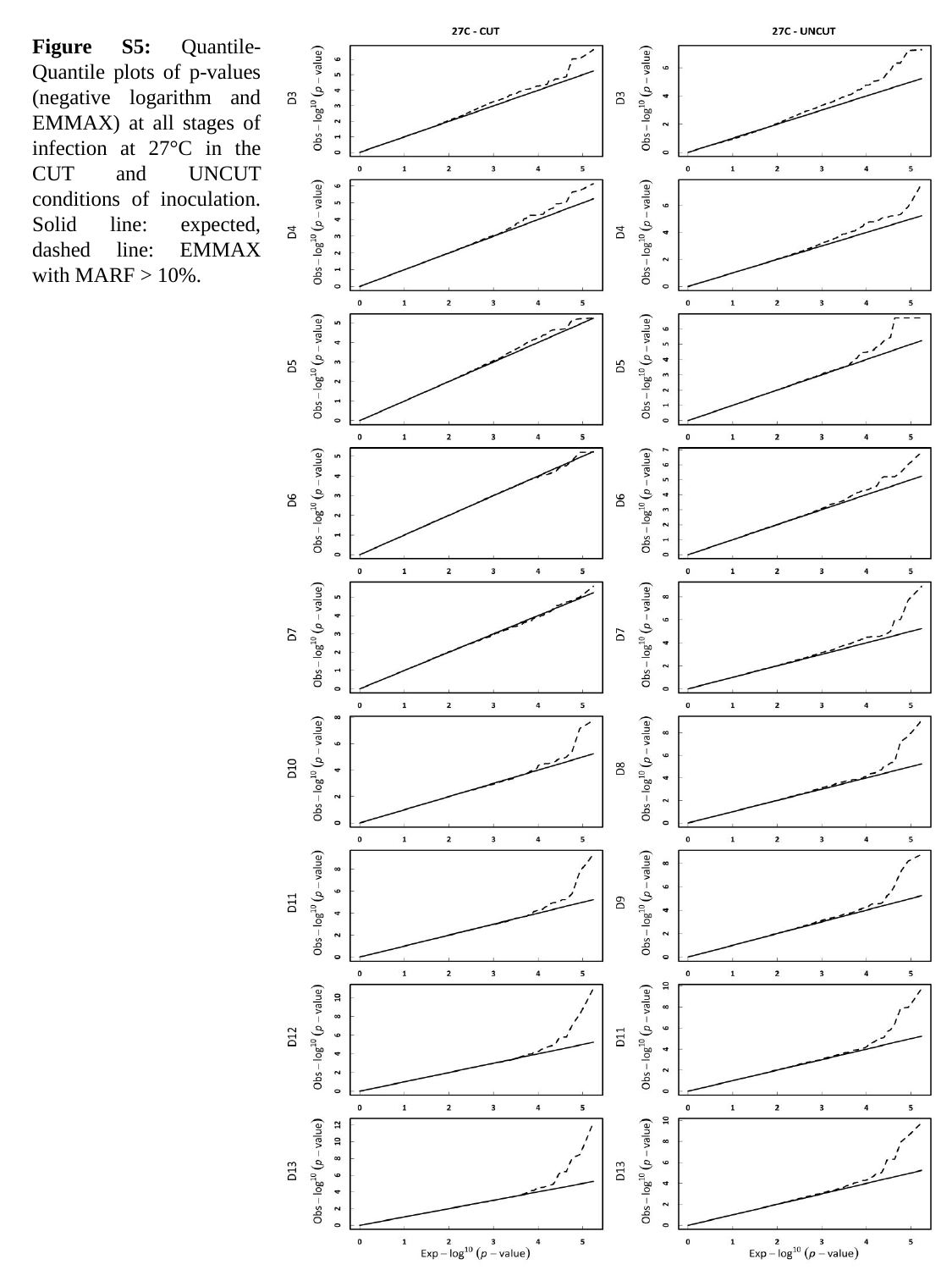

Figure S5: Quantile-Quantile plots of p-values (negative logarithm and EMMAX) at all stages of infection at 27°C in the CUT and UNCUT conditions of inoculation. Solid line: expected, dashed line: EMMAX with MARF > 10%.

## Slide 6
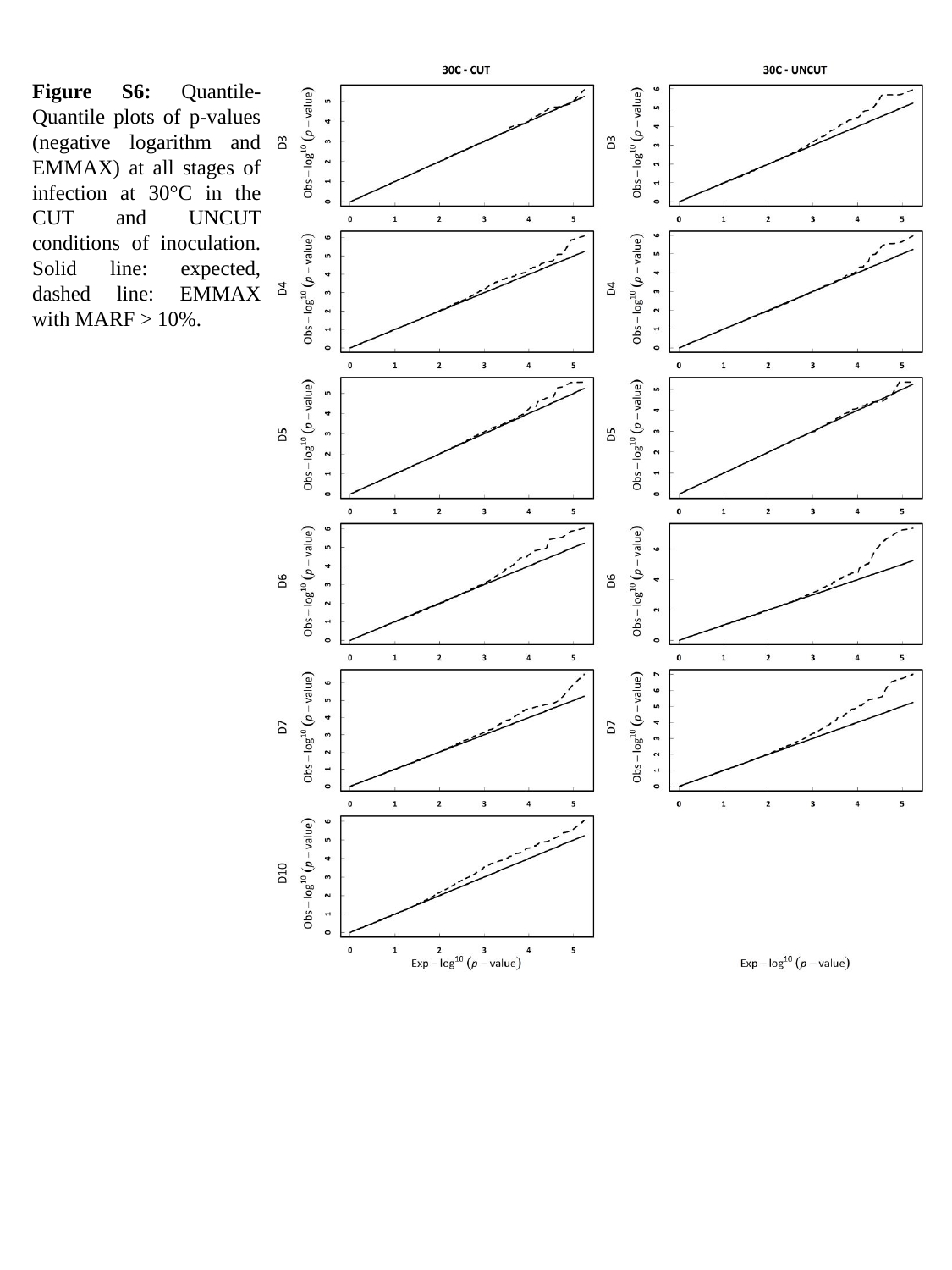

Figure S6: Quantile-Quantile plots of p-values (negative logarithm and EMMAX) at all stages of infection at 30°C in the CUT and UNCUT conditions of inoculation. Solid line: expected, dashed line: EMMAX with MARF > 10%.

## Slide 7
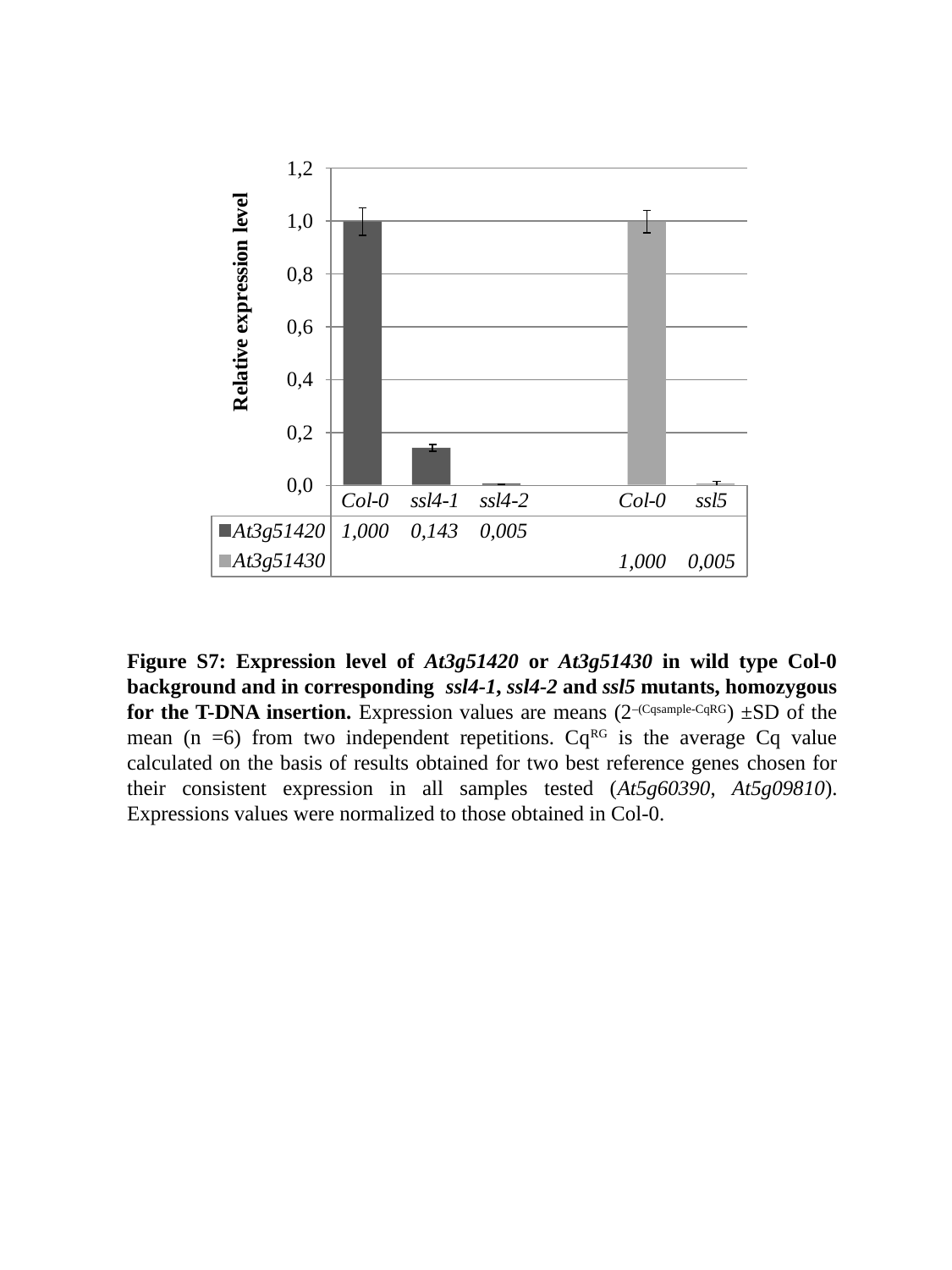

Figure S7: Expression level of At3g51420 or At3g51430 in wild type Col-0 background and in corresponding ssl4-1, ssl4-2 and ssl5 mutants, homozygous for the T-DNA insertion. Expression values are means (2–(Cqsample-CqRG) ±SD of the mean (n =6) from two independent repetitions. CqRG is the average Cq value calculated on the basis of results obtained for two best reference genes chosen for their consistent expression in all samples tested (At5g60390, At5g09810). Expressions values were normalized to those obtained in Col-0.

## Slide 8
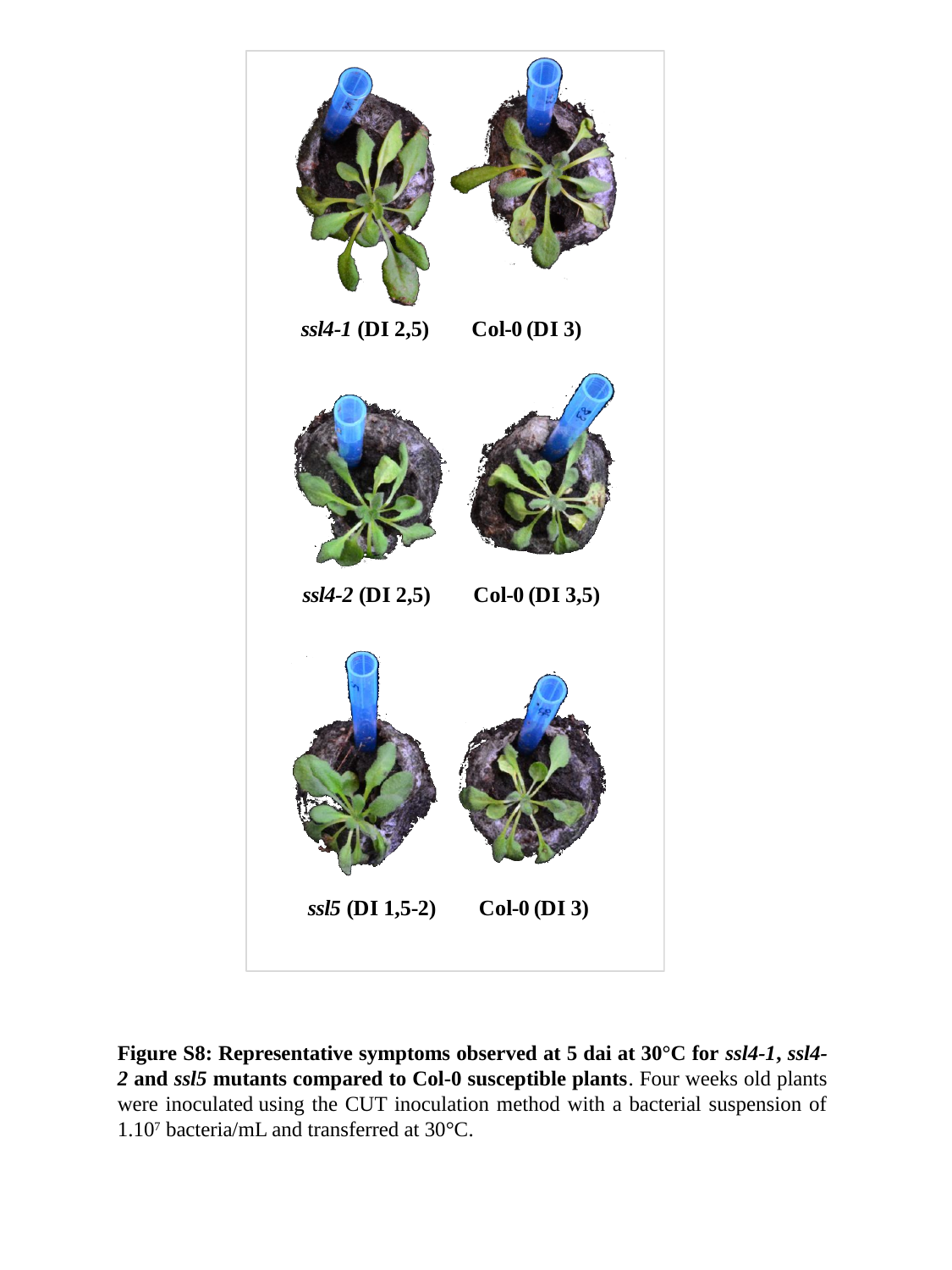

Figure S8: Representative symptoms observed at 5 dai at 30°C for ssl4-1, ssl4-2 and ssl5 mutants compared to Col-0 susceptible plants. Four weeks old plants were inoculated using the CUT inoculation method with a bacterial suspension of 1.107 bacteria/mL and transferred at 30°C.
